# Hepatocyte Nuclear Factor-1β Induces Redifferentiation of Dedifferentiated Tubular Epithelial Cells

**DOI:** 10.1371/journal.pone.0154912

**Published:** 2016-05-19

**Authors:** Mitsugu Omata, Yukiko Doke, Chikaomi Yamada, Kayoko Kawashima, Rumiko Sho, Kei Enomoto, Mayumi Furuya, Norio Inomata

**Affiliations:** Asubio Pharma Co., Ltd., Kobe, Japan; INSERM, FRANCE

## Abstract

Tubular epithelial cells (TECs) can be dedifferentiated by repetitive insults, which activate scar-producing cells generated from interstitial cells such as fibroblasts, leading to the accumulation and deposition of extracellular matrix molecules. The dedifferentiated TECs play a crucial role in the development of renal fibrosis. Therefore, renal fibrosis may be attenuated if dedifferentiated TECs are converted back to their normal state (re-epithelialization). However, the mechanism underlying the re-epithelialization remains to be elucidated. In the present study, TGF-β1, a profibrotic cytokine, induced dedifferentiation of cultured TECs, and the dedifferentiated TECs were re-epithelialized by the removal of TGF-β1 stimulation. In the re-epithelialization process, transcription factor hepatocyte nuclear factor 1, beta (HNF-1β) was identified as a candidate molecule involved in inducing re-epithelialization by means of DNA microarray and biological network analysis. In functional validation studies, the re-epithelialization by TGF-β1 removal was abolished by HNF-1β knockdown. Furthermore, the ectopic expression of HNF-1β in the dedifferentiated TECs induced the re-epithelialization without the inhibition of TGF-β/Smad signaling, even in the presence of TGF-β1 stimulation. In mouse renal fibrosis model, unilateral ureteral obstruction model, HNF-1β expression in the TECs of the kidney was suppressed with fibrosis progression. Furthermore, the HNF-1β downregulated TECs resulted in dedifferentiation, which was characterized by expression of nestin. In conclusion, HNF-1β suppression in TECs is a crucial event for the dedifferentiation of TECs, and the upregulation of HNF-1β in TECs has a potential to restore the dedifferentiated TECs into their normal state, leading to the attenuation of renal fibrosis.

## Introduction

Tissue fibrosis, which is a pathological scarring process associated with the relentless production and deposition of extracellular matrix molecules, including collagen and fibronectin, is a leading cause of dysfunction and failure in many organs [1]. Approximately 45% of all deaths in developed western countries are reportedly attributable to fibrotic diseases [2]. Despite this unmet medical need, there are no established therapies for tissue fibrosis. Chronic kidney disease (CKD) is a global public health problem, and its prevalence is estimated to be 8%–16% worldwide [3]. Tubulointerstitial fibrosis represents a common feature of CKD, regardless of the primary underlying disease [4,5]. A number of studies have shown that the histologic severity of interstitial fibrosis is correlated with kidney function decline in patients with CKD [6–8]. The onset of tubulointerstitial fibrosis is caused by damage of the tubular epithelial cells (TECs), which first encounters various stimuli, including inflammation, ischemia, infection, toxins, and the profibrotic cytokine TGF-β [9,10]. The damaged TECs undergo a dedifferentiation, which is characterized by loss of cytoskeletal integrity, cell polarity and epithelial marker molecules (γ-glutamyl transferase [γ-GT], claudin-2, and E-cadherin), and acquisition of mesenchymal marker molecules (vimentin, fibronectin, type I collagen and nestin) [11–13]. With short-term injuries, the dedifferentiated TECs proliferate and re-differentiate to restore the functional and structural integrity of nephrons [14]. However, when TECs remain in the dedifferentiation state due to severe and repetitive stimulation, the dedifferentiated cells release profibrotic mediators that can induce the generation of scar-producing cells from various tubulointerstitial cells, including fibroblasts and pericytes, leading to the accumulation and deposition of extracellular matrix molecules and the development of renal fibrosis [9,11,12,15]. The dedifferentiated TECs also produce inflammatory molecules such as chemokines and active lipids, recruiting inflammatory cells to the tubulointerstitium, and these inflammatory cells, such as monocytes/macrophages, T cells and dendritic cells, contribute to the pathogenesis of renal fibrosis [11,12,16]. Furthermore, the activated scar-producing cells and inflammatory cells potentiate the dedifferentiated TECs, creating a vicious cycle of renal fibrosis. Recently, it was reported that the microenvironment of fibrotic disease contributes to the progression of tissue fibrosis [17]. As most of the tubulointerstitial structure is occupied by TECs, it is believed that the phenotype or state of TECs are major factors in determining the creation and maintenance of the fibrotic microenvironment. Accordingly, the conversion of dedifferentiated TECs back to their normal state (re-epithelialization) may induce a normal microenvironment in the kidney, leading to quiescence of activated scar-producing cells and inflammatory cells, thus ameliorating renal fibrosis. However, the mechanism underlying the normalization of dedifferentiated TECs remains to be elucidated.

In the present study, we show that TGF-β1 induced dedifferentiation of cultured human renal proximal tubular epithelial cells (hRPTECs) and that the dedifferentiated hRPTECs were re-epithelialized by the removal of TGF-β1 stimulation. We also showed that a transcription factor hepatocyte nuclear factor 1, beta (HNF-1β) restored TGF-β1-induced dedifferentiated hRPTECs to their normal status using a DNA microarray system, biological network analysis and functional validation of candidate genes for re-epithelialization. Furthermore, the expression of HNF-1β in TECs was downregulated in association with the development of renal fibrosis in *in vivo* model. To the best of our knowledge, this is the first report that shows the possibility of a new therapeutic strategy for renal fibrosis based on the re-epithelialization effect of HNF-1β.

## Materials and Methods

### Induction of re-epithelialization of TGF-β1-stimulated hRPTECs by removal of TGF-β1

hRPTECs were purchased from Lonza (CC-2553; Basel, Switzerland), and were used between the fifth and seventh passages in renal epithelial basal medium supplemented with REGM Singlequots Bulletkit (CC-3190; Lonza). These cells were seeded onto collagen I-coated six-well plates at a density of 3 × 10^5^ cells/well and cultivated until sub-confluent, and the medium was then replaced with serum-free medium for starvation. After starvation for 24 h, the hRPTECs were stimulated to dedifferentiate by 3 ng/mL of TGF-β1 for 48 h; thereafter, re-differentiation (re-epithelialization) was induced by incubation with TGF-β1-free fresh medium for 48 h, 72 h, or 96 h.

### Treatment of siRNA in the re-epithelialization of TGF-β1-stimulated hRPTECs by removal of TGF-β1

The hRPTECs were seeded onto collagen I-coated six-well plates at a density of 3 × 10^5^ cells/well and cultivated until sub-confluent, and the medium was then replaced with serum-free medium for starvation. After starvation for 24 h, the hRPTECs were stimulated with 3 ng/mL of TGF-β1 for 48 h, followed by incubation in fresh medium with or without TGF-β1 for 48–96 h. Cells were treated with three types of siRNA (15 nM) against HNF1A, HNF1B, HNF4A, LGALS2, GDF15, NAT8, EPHA7, SLCO4A1, NR1H4, and MITF or two types of negative control siRNA (Silencer® Select siRNA; Life Technologies, Carlsbad, CA, USA) (15 nM) 24 h before the removal of the TGF-β1.

### DNA microarray analysis

Gene expression profiling of the dedifferentiated hRPTECs that were stimulated by TGF-β1 for 48 h (n = 2) and followed by additional TGF-β1 stimulation for 24 h (n = 1) and the re-epithelialized hRPTECs by TGF-β1 removal (n = 1) was investigated by DNA microarray. Briefly, total RNA was purified using an RNeasy Kit (Qiagen, Hamburg, Germany) following the manufacturer’s instructions. Cy3-labeled complementary RNA (cRNA) was obtained from 200 ng of total RNA using the Agilent Low Input Quick Amp Labeling Kit (Agilent Technologies, Santa Clara, CA, USA). Cy3-labeled cRNA was hybridized to the Whole Human Genome Oligo Microarray ver. 2.0 (4 × 44 k) (Agilent Technologies) following the manufacturer’s hybridization protocol. After the washing step, the microarray slides were analyzed using the Agilent Microarray Scanner B version (Agilent Technologies) with the default settings for all parameters. Microarray expression data were obtained using Agilent Feature Extraction software ver. 10.5.1 (Agilent Technologies) with the default settings for all parameters. The analyses of the datasets were performed on Subio Platform ver. 1.16 and Subio Basic Plug-in program (Subio, Tokyo, Japan). Functional clustering and network analysis of the genes extracted from the dataset analyses were performed using Ingenuity Pathway Analysis (IPA; Ingenuity Systems, Redwood City, CA, USA).

### Preparation of recombinant adenovirus expressing HNF1B (Ad-HNF1B)

To generate Ad-HNF1B, a cDNA fragment encoding human HNF1B was amplified using PCR and cloned into the *Swa*I site of the cosmid vector pAxCAWtit2 (Takara Bio, Shiga, Japan). The oligonucleotide sequences specific for human HNF1B are: 5ʹ-AGCGATATCATGGTGTCCAAGCTCACGTCGCT-3ʹ (sense) and 5ʹ-AGCGATATCTCACCAGGCTTGTAGAGGACA-3ʹ (antisense).

Recombinant adenoviruses were generated by the full-length DNA transfer method in 293 cells using the Adenovirus Expression Vector Kit (Dual Version) ver. 2 (Takara Bio) according to the manufacturer’s instructions.

### Re-epithelialization of dedifferentiated hRPTECs by adenovirus-mediated ectopic expression of HNF1B

The hRPTECs were seeded onto collagen I-coated six-well plates at a density of 3 × 10^5^ cells/well and cultivated until sub-confluent, and then the cultured medium was replaced with serum-free medium for starvation. After starvation for 24 h, the hRPTECs were stimulated with 3 ng/mL of TGF-β1 for 48 h, and thereafter, re-stimulated with 3 ng/mL of fresh TGF-β1. After replacement with fresh TGF-β1, the hRPTECs were infected with 0.1, 0.3, 0.5, 1, 2, and 3 MOI (multiplicity of infection; infection of one cell with one virus particle is indicated by MOI = 1) of HNF1B or LacZ-expressed adenovirus vector. After one day of infection, the medium was replaced with fresh TGF-β1. The epithelial cells were collected 72 h after infection for gene expression analysis. To detect any morphological phenotype changes, 96 h after infection the epithelial cells were fixed with 4% paraformaldehyde (PFA) for 15 min at room temperature. After permeabilization with 0.1% Triton X 100, the cells were incubated with 1% bovine serum albumin for 30 min, followed by incubation with Alexa Fluor® 594 phalloidin (1:20 dilution; Life Technologies) for 20 min. After washing, the cells were mounted with SlowFade® Gold Antifade Reagent (Life Technologies).

### Real-time RT-PCR

Total RNA was isolated using a commercial kit (RNase mini kit; Qiagen). cDNA was generated from 1.5 μg of total RNA and oligo(dT) primers using the SuperScript First-Strand Synthesis System (Life Technologies) for RT-PCR according to the manufacturer’s protocol. Quantitative real-time TaqMan PCR was performed in a 40-μL reaction volume containing 1 μL of cDNA template, 20 μL of TaqMan® Universal Master Mix (Life Technologies), using TaqMan® Gene Expression Assays consisting of a pair of unlabeled PCR primers and a TaqMan probe with a FAM dye label (Life Technologies). The PCR amplification profiles included an initial incubation at 50°C for 2 min, denaturation at 95°C for 10 min, and 40 to 45 cycles of 95°C for 15 s and 60°C for 1 min. All reactions were performed in duplicate, and all data were presented after normalization to the GAPDH or the 18S ribosomal RNA expression observed in the same sample.

### Western blot analysis

The expression levels of HNF-1β, phosphorylated Smad3, and total Smad3 protein were examined by western blotting. Briefly, the hRPTECs and mouse kidneys were lysed in 0.5 ml of ice-cold lysis buffer. The samples were centrifuged at 12,000 × *g* for 10 min, and the supernatants were used for the assay. After mixing with SDS-PAGE sample buffer and boiling for 5 min, the samples (20 μg/lane) were electrophoresed on 7.5% SDS polyacrylamide gels and transferred to polyvinylidene difluoride membranes for 1.5 h at 180 mA. The membranes were blocked for 1 h with Block-Ace (DS Pharma Biomedical, Osaka, Japan) and incubated with primary anti-HNF-1β polyclonal antibody (1:1600 dilution; Proteintech Group, Chicago, IL, USA), anti-Smad3 phospho (Ser423/425) monoclonal antibody (1:1000 dilution; ab52903; Abcam, Cambridge, UK), and anti-Smad3 monoclonal antibody (1:5000 dilution; ab40854; Abcam) overnight at 4°C. After washing, the membranes were incubated with HRP-conjugated anti-rabbit IgG for 1 h. The western blots were visualized using the enhanced chemiluminescence system (LumiGLO Reagent; Cell Signaling Technology, Danvers, MA, USA). The same membranes were stripped and reprobed with anti-β-actin antibody (1:200 dilution; Santa Cruz Biotechnology, Dallas, TX, USA) to confirm equal loading. Densitometric quantification of the corresponding bands was performed using an image analyzer (BAS3000; Fuji Photo Film, Tokyo, Japan). The data were presented after normalization to β-actin expression.

### Induction of renal fibrosis model in mice

All animal experiments were approved by the Animal Care and Ethics Committee of Asubio Pharma Co., Ltd. The animals were quarantined for 1 week in the animal room assigned for the study and only those without any abnormal findings at the end of this acclimation period were selected for experimentation. All surgical procedures were performed under anesthesia to minimize pain and distress in the animals. Humane endpoints were applied for animal which became severely ill or moribund via early euthanasia prior to the experimental endpoints. The animals were monitored for physical condition daily to the termination of the experiment. Although criteria (including inability to access food or water, rapid loss of more than 20% of body weight within a few days, labored respiration) for early euthanasia were in place, early euthanasia was not necessary. Renal fibrosis models, namely unilateral ureteral obstruction (UUO), were induced in female ICR mice (7 weeks old; Charles River Japan, Kanagawa, Japan) as previously described [18]. In the UUO mice, the left ureter was exposed through a flank incision and ligated with 4–0 silk sutures at two points under anesthesia with isoflurane inhalation. After 3, 5, 7 or 10 days, the kidneys were collected and bisected longitudinally for protein expression and histological studies. Half of each kidney was immediately frozen in liquid nitrogen and stored at −80°C until further analysis. The remainder was immersed in 4% PFA for histologic examination.

### Histologic examination

The PFA-fixed kidneys from the UUO mice were embedded in paraffin, sectioned into 5-μm thick slices, and stained with Masson’s trichrome reagents. The sections were evaluated by an independent observer blinded to the origin of the slides. For quantification of tubulointerstitial fibrosis, 20 fields were randomly selected from the cortical region and were analyzed under high-power magnification (×400). Fibrotic areas in the interstitium, which were stained blue, were identified on the digital images using a computer-aided manipulator (LuminaVision; Mitani, Fukui, Japan). The percentage of the fibrotic area was calculated. Glomeruli and large vessels were excluded from the analysis.

### Immunohistochemistry

To examine the localization of HNF-1β within the kidneys, double immunofluorescence staining of renal tissue was performed using anti-HNF-1β antibody and anti-nestin (mesenchymal marker) antibody. For double staining of the anti-HNF-1β antibody and anti-nestin antibody, slides with 5-μm deparaffinized tissue sections were placed in 0.01 M citrate and heated twice in a microwave oven at 2450 MHz and 800 W for 7 min each. The slides were incubated in normal goat blocking serum for 30 min, followed by overnight incubation at 4°C with polyclonal rabbit antibody against HNF-1β (HPA002083; Sigma-Aldrich) and monoclonal mouse antibody against nestin (MAB353; Merck Millipore, Billerica, MA, USA). After washing, Alexa Fluor® 594 goat anti-rabbit antibody (1:500 dilution; A-11012; Life Technologies) and Alexa Fluor® 488 goat anti-mouse antibody (1:500 dilution; A-11001; Life Technologies) were added for 60 min at room temperature. After washing, the sections were mounted with SlowFade® Gold Antifade Reagent (Life Technologies).

### Statistical analyses

Comparisons between two groups were performed using either *t*-tests or Wilcoxon’s rank-sum tests when the data were normally distributed or the observations showed considerable variability, respectively. Differences among three or more groups were evaluated using two-way analysis of variance, followed by Dunnett’s test. *P* < 0.05 was considered to be statistically significant.

## Results

### Re-epithelialization of dedifferentiated hRPTECs by TGF-β1 removal

**Fig 1:**
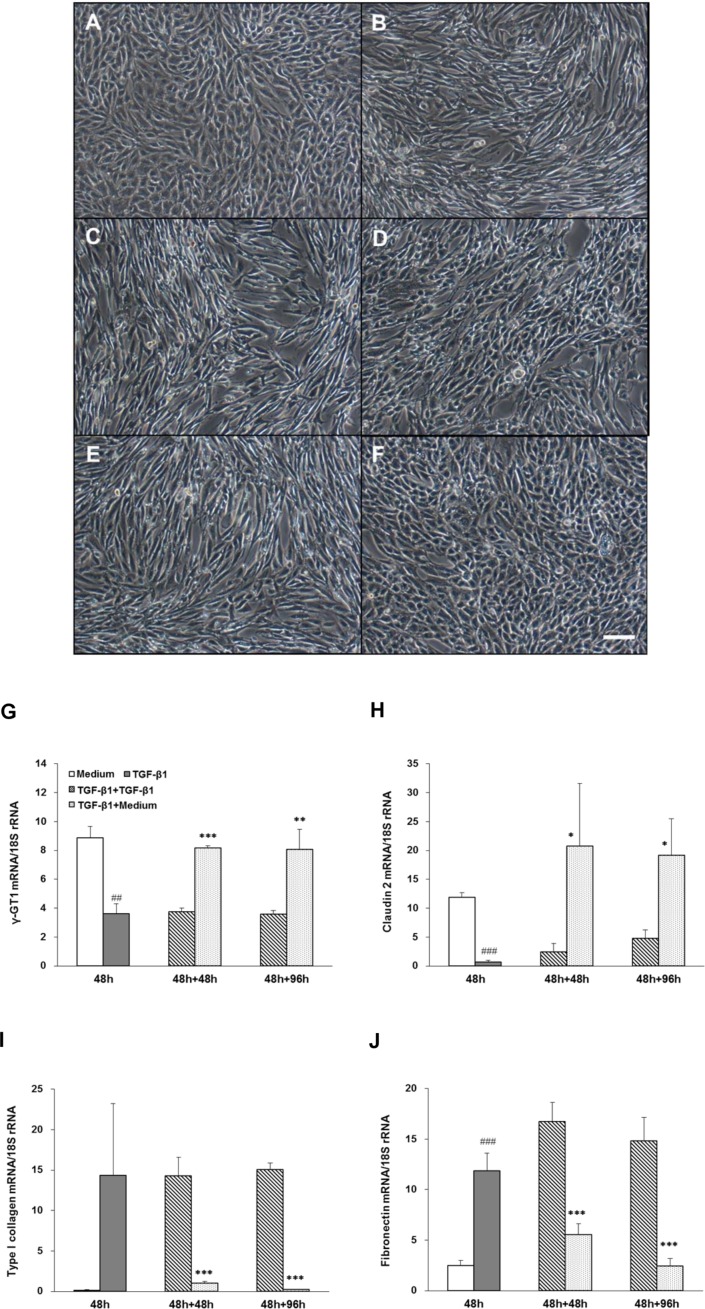
Time course of morphological changes and gene expression in re-epithelialized hRPTECs induced by TGF-β1 removal. Human RPTECs were cultivated with medium or 3 ng/ml TGF-β1 for 48 h, followed by incubation in fresh medium with or without TGF-β1 for 48 h or 96 h. Representative phase-contrast microscopy photographs of hRPTECs show non-stimulation (A), TGF-β1 stimulation for 48 h (B), TGF-β1 stimulation for 48 h followed by TGF-β1 re-stimulation for 48 (C) or 96 h (E), and TGF-β1 stimulation for 48 h followed by incubation with TGF-β1-free fresh medium for 48 (D) or 96 h (F). Scale bar = 100 μm. The levels of mRNA encoding proximal tubular epithelial marker genes (G: γ-glutamyl transferase [γ-GT1] and H: claudin-2) and mesenchymal marker genes (I: type I collagen and J: fibronectin) in differentiated hRPTECs were determined by real-time RT-PCR analyses. Each column shows data from non-stimulation (white), TGF-β1 stimulation for 48 h (gray), TGF-β1 stimulation for 48 h followed by TGF-β1 re-stimulation (hatched line), and TGF-β1 stimulation for 48 h followed by incubation with TGF-β1-free fresh medium (dot). Each column and bar presents the means ± SD of three independent experiments. Statistical significance: ## *P* < 0.01, ### *P* < 0.001 vs. medium group (white); * *P* < 0.05, ** *P* < 0.01, *** *P* < 0.001 vs. TGF-β1 re-stimulation group (hatched line) at each time point by *t*-tests.

Treatment with TGF-β1 for 48 h induced dedifferentiation of hRPTECs, which was characterized by a change in cell morphology from a cobblestone appearance (Fig 1A) to a spindle shape (Fig 1B), suppression of proximal tubular epithelial cell marker genes such as γ-GT1 and claudin-2 mRNA (Fig 1G and 1H), and upregulation of mesenchymal marker genes such as type I collagen and fibronectin mRNA (Fig 1I and 1J) in hRPTECs. In the presence of TGF-β1, these changes in cell morphology and marker genes lasted for at least 48 h (Fig 1C) or 96 h (Fig 1E). Removal of TGF-β1 from the culture medium for 48 h or 96 h following TGF-β1 stimulation for 48 h resulted in the dedifferentiated hRPTECs being restored to their normal epithelial morphology (Fig 1D and 1F), and both the epithelial and mesenchymal marker expression were also normalized at 48 h and 96 h (Fig 1G–1J). Therefore, the genes/factors that play crucial roles in inducing the re-epithelialization were identified using this same re-epithelialization system by removing TGF-β1.

### Identification of candidate genes involved in re-epithelialization

Gene expression profiling of the dedifferentiated hRPTECs that were stimulated by TGF-β1 for 48 h (Group 1) and followed by additional TGF-β1 stimulation for 24 h (Group 2) and the re-epithelialized hRPTECs by TGF-β1 removal (Group 3) was investigated by DNA microarray. Comparative differential gene expression analysis among these three groups using the Subio Platform revealed that 872 genes were downregulated in Group 2 compared with Group 1 and were upregulated more than two-fold in Group 3 compared with Group 1 (S1 Table). The biological networks of the genes altered during re-epithelialization were analyzed using IPA, and 48 genes that were located in the central position of 25 networks generated through IPA were selected as candidate genes involved in re-epithelialization (S1 Fig, S2 Table). We expected that the candidate genes involved in inducing the re-epithelialization may be upregulated a short period after TGF-β1 removal. Therefore, the expression changes of 48 candidate genes within 24 h were measured using real-time RT-PCR. The expression of several candidate genes, including HNF1A, HNF1B, HNF4A, LGALS2, GDF15, NAT8, ELF3, CEBPA, and NR1H4 began to increase within 6 h after TGF-β1 removal (S2 Fig).

### Functional validation of candidate genes for re-epithelialization

**Fig 2:**
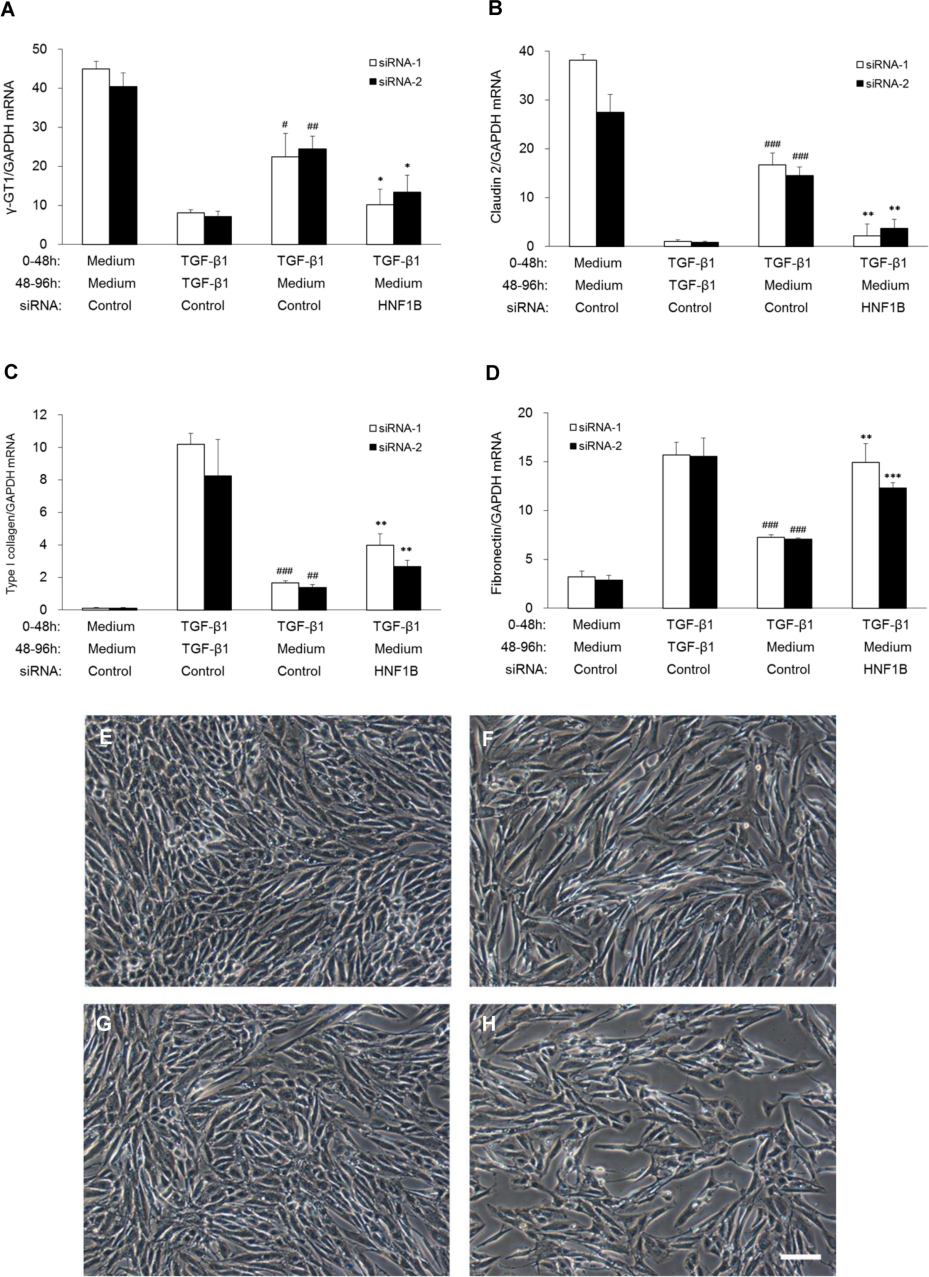
Effect of HNF1B-targeting siRNA on re-epithelialization (gene expression and morphological changes) by TGF-β1 removal. Human RPTECs were cultivated with medium or 3 ng/ml TGF-β1 for 48 h, followed by incubation in fresh medium with or without TGF-β1 for 48 h (gene expression experiment: A–D) and 96 h (morphology experiment: E–H). Cells were treated with two types of HNF1B-targeting siRNA (15 nM) and negative control siRNA (Control) (15 nM) for 24 h after the first TGF-β1 stimulation. The levels of mRNA encoding proximal tubular epithelial marker genes (A: γ-GT1 and B: claudin-2) and mesenchymal marker genes (C: type I collagen and D: fibronectin) in differentiated hRPTECs were determined by real-time RT-PCR. Each column shows data from siRNA-1 (white) and siRNA-2 (black). Each column and bar presents the means ± SD of three independent experiments. Statistical significance: # P < 0.05, ## P < 0.01, ### P < 0.001 vs. TGF-β1 (0-48h) + TGF-β1 (48-96h) with corresponding Control siRNA; * P < 0.05, ** P < 0.01, *** P < 0.001 vs. TGF-β1 (0-48h) + Medium (48-96h) with corresponding Control siRNA by t-tests. Representative phase-contrast microscopy photographs of the hRPTECs show non-stimulation (E), TGF-β1 stimulation for 48 h (F), TGF-β1 stimulation for 48 h followed by incubation with TGF-β1-free fresh medium with control siRNA (G) and HNF1B siRNA (H) for 96 h. Scale bar = 100 μm.

To evaluate the ability of the candidate genes to induce re-epithelialization, we investigated the knockdown effects of the candidate genes on the epithelialization induced by TGF-β1 removal. Although the suppression of the epithelial gene expression and the upregulation of the mesenchymal gene expression in TGF-β1-induced dedifferentiated hRPTECs were restored to the normal hRPTEC level by TGF-β1 removal, the restoration was inhibited by the treatment of siRNA targeting HNF1B. However, any of the siRNAs for the other candidate genes did not affect the expression change of the epithelial and mesenchymal marker genes during the re-epithelialization (S3 Fig). The expression of endogenous HNF1B was suppressed in the dedifferentiated hRPTECs and recovered to the normal level after TGF-β1 removal. The upregulation was silenced with three types of siRNAs (siRNA-1, -2, and -3) targeting HNF1B by 81%, 46%, and 31%, respectively, compared with TGF-β1 removal and the control siRNA-treated group (S4B Fig). The inhibitory activity of the three types of HNF1B siRNAs on re-epithelialization was closely correlated with their silencing strength for endogenous HNF1B gene expression. In particular, HNF1B-targeting siRNA-1, which exhibited the strongest silencing effect on the endogenous gene expression, remarkably abolished the recovery of the epithelial and mesenchymal gene expression. To reconfirm the participation of HNF1B in the re-epithelialization, we investigated the effect of HNF1B knockdown using two types of HNF1B-targeting siRNAs (siRNA-1 and -2), which are the same with the siRNAs presented in the candidate genes validation study (S3 Fig). Both siRNAs significantly abrogated not only the upregulation of the epithelial gene expression (Fig 2A and 2B) and the suppression of the mesenchymal gene expression (Fig 2C and 2D), but also the cell morphology change from a spindle shape (Fig 2F) to a cobblestone appearance (Fig 2G) as induced by TGF-β1 removal (Fig 2H). These results suggest that HNF1B was strongly involved in the induction of the re-epithelialization of hRPTECs by TGF-β1 removal.

### Induction of re-epithelialization by ectopic HNF-1β expression

**Fig 3:**
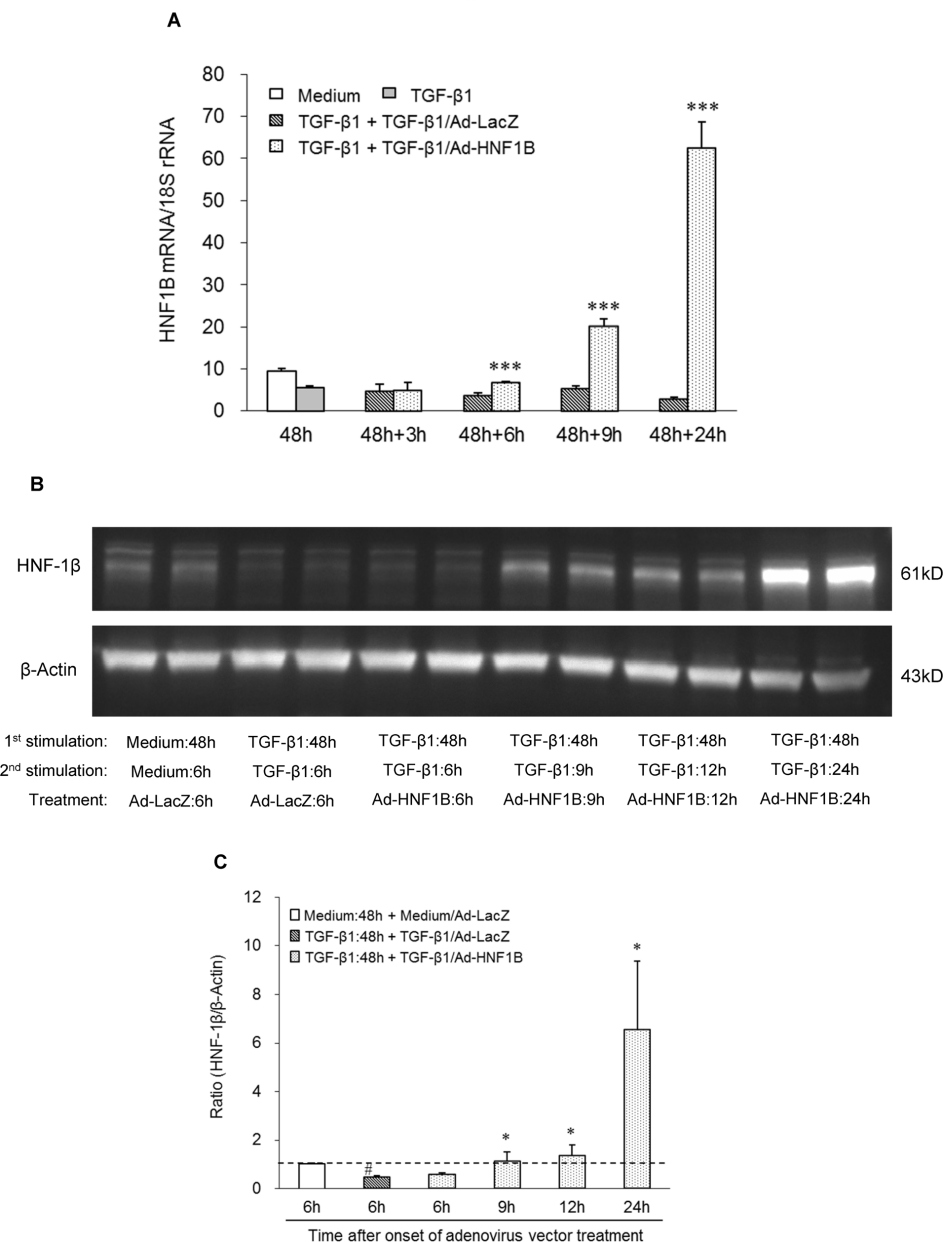
Time course of HNF1B gene and protein expression in differentiated hRPTECs after Ad-HNF1B infection. Human RPTECs were cultivated with medium or 3 ng/ml TGF-β1 for 48 h, followed by re-stimulation with TGF-β1 for 24 h. After TGF-β1 stimulation for 48 h, the hRPTECs were infected with Ad-HNF1B or Ad-LacZ. The expression level of HNF1B mRNA was determined by real-time RT-PCR analysis (A). Each column shows data from non-stimulation (white), TGF-β1 stimulation for 48 h (gray), TGF-β1 stimulation for 48 h followed by TGF-β1 re-stimulation with Ad-LacZ (hatched line), and with Ad-HNF1B (dot) for 3, 6, 9 and 24 h. Each column and bar presents the means ± SD of three independent experiments. Statistical significance: *** P < 0.001 vs. corresponding TGF-β1 and Ad-LacZ-treated groups (hatched column) by t-tests. The expression of HNF1B protein (HNF-1β) 6, 9, 12, and 24 h after adenovirus infection was analyzed using the western blot method. A representative photograph shows the expression of HNF-1β at 61 kD, with β-actin at 43 kD as a control, in adenovirus vector-infected hRPTECs (B). Densitometric quantification of the corresponding bands was performed using an image analyzer. The data were presented after normalization to β-actin expression (C). Each column shows data from non-stimulation with Ad-LacZ (white), TGF-β1 stimulation for 48 h followed by incubation with TGF-β1 and Ad-LacZ for 6 h (hatched line), and TGF-β1 stimulation for 48 h followed by incubation with TGF-β1 and Ad-HNF1B for 6, 9, 12, and 24 h (dot). Each column and bar presents the means ± SD of three independent experiments. The dotted line indicates the protein expression at 6 h in the non-stimulation with Ad-LacZ group. Statistical significance: # *P* < 0.05 vs. non-stimulation with Ad-LacZ group (white); * P < 0.05 vs. TGF-β1 stimulation with Ad-LacZ group (hatched line) by t-tests.

**Fig 4:**
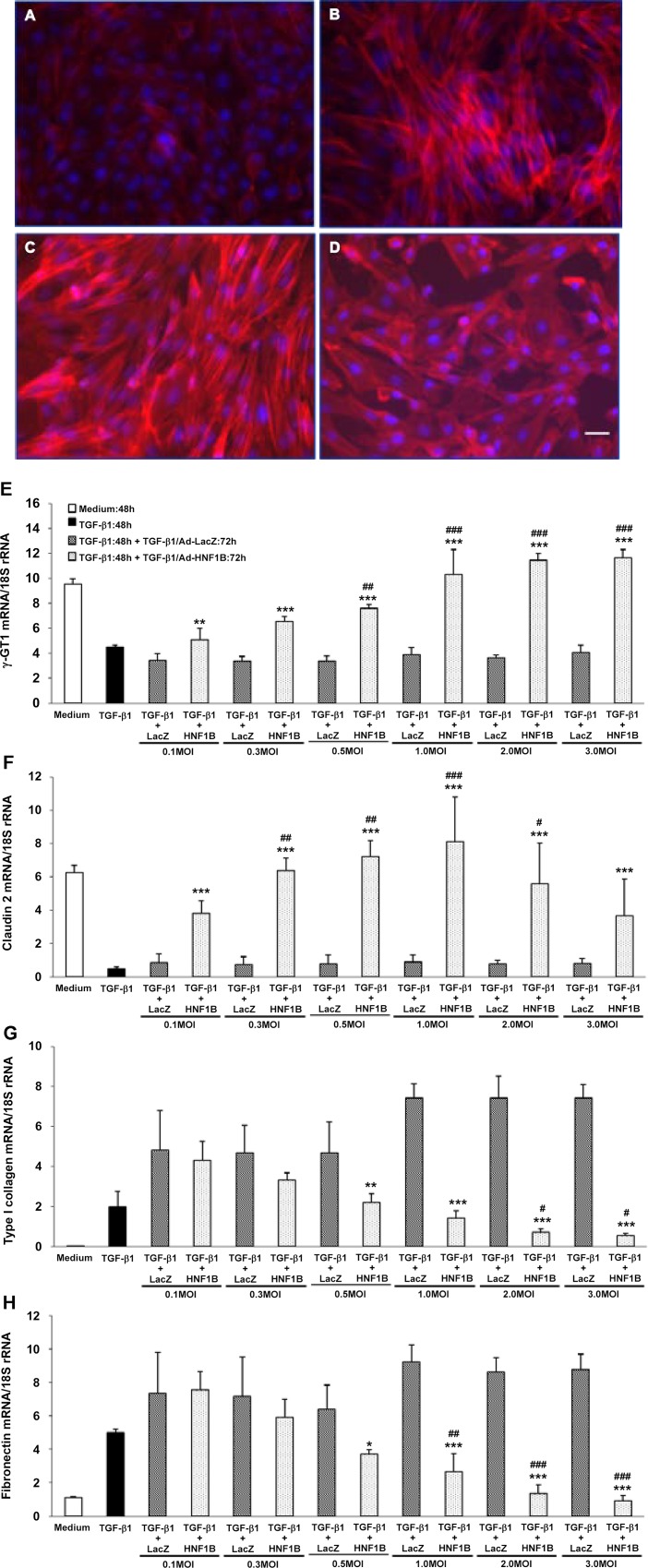
Effect of HNF-1β ectopic expression on morphological changes and gene expression of dedifferentiated hRPTECs. Human RPTECs were stimulated with 3 ng/ml TGF-β1 for 48 h followed by re-stimulation with fresh TGF-β1 for 72 h. After replacement with fresh TGF-β1, the hRPTECs were infected with 3.0 MOI Ad-HNF1B or Ad-LacZ. Representative fluorescence microscopy photographs of hRPTECs show non-stimulation (A), TGF-β1 stimulation for 48 h (B), TGF-β1 stimulation for 48 h followed by treatment with TGF-β1 and 3.0 MOI of Ad-LacZ for 72 h (C), and TGF-β1 stimulation for 48 h followed by treatment with TGF-β1 and 3.0 MOI of Ad-HNF1B for 72 h (D). Scale bar = 30 μm. Human RPTECs were stimulated with 3 ng/ml TGF-β1 for 48 h, followed by re-stimulation with fresh TGF-β1 for 72 h. After replacement with fresh TGF-β1, the hRPTECs were infected with 0.1 to 3.0 MOI Ad-HNF1B or Ad-LacZ. The levels of mRNA encoding γ-GT1 (E), claudin-2 (F), type I collagen (G), and fibronectin (H) in the differentiated hRPTECs were determined by real-time RT-PCR. Each column shows the data from non-stimulation (white), TGF-β1 stimulation for 48 h (black), TGF-β1 stimulation for 48 h followed by treatment with TGF-β1 and Ad-LacZ for 72 h (hatched line), and TGF-β1 and Ad-HNF1B for 72 h (dot). Each column and bar presents the mean ± SD from three independent experiments. Statistical significance: # *P* < 0.05, ## *P* < 0.01, ### *P* < 0.001 vs. 48-h TGF-β1-treated group (black column); ** *P* < 0.01, *** *P* < 0.001 vs. corresponding TGF-β1- and Ad-LacZ-treated groups (hatched column) by *t*-tests.

**Fig 5:**
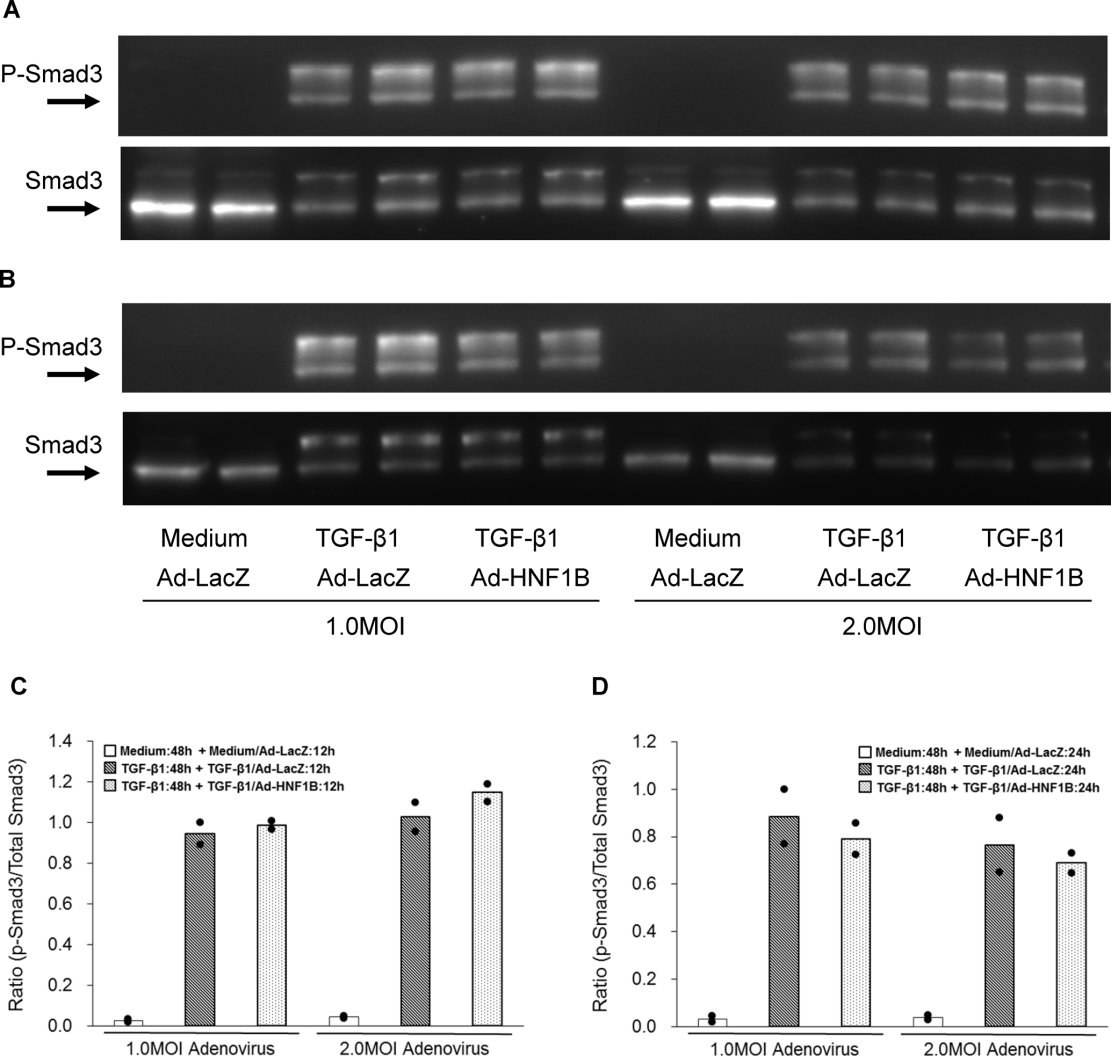
Effect of HNF-1β ectopic expression on TGF-β1-induced phosphorylation of Smad3 in hRPTECs. Human RPTECs were stimulated with 3 ng/ml TGF-β1 for 48 h followed by re-stimulation with fresh TGF-β1 for 12 h or 24 h. After replacement with fresh TGF-β1, the hRPTECs were infected with 1.0 MOI and 2.0 MOI Ad-HNF1B or Ad-LacZ. Representative western blotting shows the expression of phosphorylated Smad3 and total Smad3 at 48 kD in Ad-HNF1B-transfected hRPTECs 12 (A) and 24 h (B) after TGF-β1 re-stimulation. Densitometric quantification of the corresponding bands was performed using an image analyzer. The data are presented after normalization to total Smad3 expression. Each column presents the means of twice independent experiments from non-stimulation with Ad-LacZ (white), TGF-β1 stimulation for 48 h followed by incubation with TGF-β1 and Ad-LacZ (hatched line), and TGF-β1 and Ad-HNF1B (dot) for 12 (C) and 24 h (D). Each dot symbol shows an individual value.

To directly evaluate the role of HNF-1β in the induction of the re-epithelialization, we investigated the re-epithelialization of dedifferentiated hRPTECs by ectopic HNF-1β expression using an adenovirus vector (Ad-HNF1B). Both HNF1B mRNA and HNF-1β protein in the hRPTECs were downregulated by TGF-β1 stimulation for 48 h. The downregulated HNF1B gene and HNF-1β recovered to the basal levels at 6 h (Fig 3A) and 9 h after Ad-HNF1B infection, respectively; furthermore, they had increased remarkably 24 h after infection (Fig 3B and 3C). TGF-β1 induced both a cell morphology change from a cobblestone appearance to a spindle shape and upregulation of stress fibers stained red by Alexa Fluor® 594 phalloidin in the hRPTECs (Fig 4B and 4C). These phenotype changes were restored by Ad-HNF1B infection 48 h after TGF-β1 stimulation (Fig 4D). The suppression of the epithelial marker genes and the increase of the mesenchymal marker genes were induced by TGF-1β stimulation for 48 h. The gene expression profiles in the dedifferentiated hRPTECs were significantly restored to their normal states by ectopic HNF1B expression, which was induced by more than 0.5 MOI of Ad-HNF1B, although TGF-β1 stimulation continued (Fig 4E–4H). Low doses (0.1 MOI and 0.3 MOI) of Ad-HNF1B recovered epithelial gene expression to their normal levels more effectively than that for the mesenchymal gene expression (Fig 4E–4H). The re-epithelialization activity of Ad-HNF1B was completely abolished by the treatment of siRNA-1 targeting HNF1B (S5 Fig). Furthermore, the effect of Ad-HNF1B on TGF-β/Smad signaling was evaluated. Although phosphorylation of Smad3 was enhanced by re-stimulation of TGF-β1 for 12 h and 24 h following the incubation with TGF-β1 for 48 h, the phosphorylation was not affected by Ad-HNF1B treatment (Fig 5). These results suggest that the ectopic HNF1B expression induced the re-epithelialization of the dedifferentiated hRPTECs without inhibiting TGF-β/Smad signaling.

**Fig 6:**
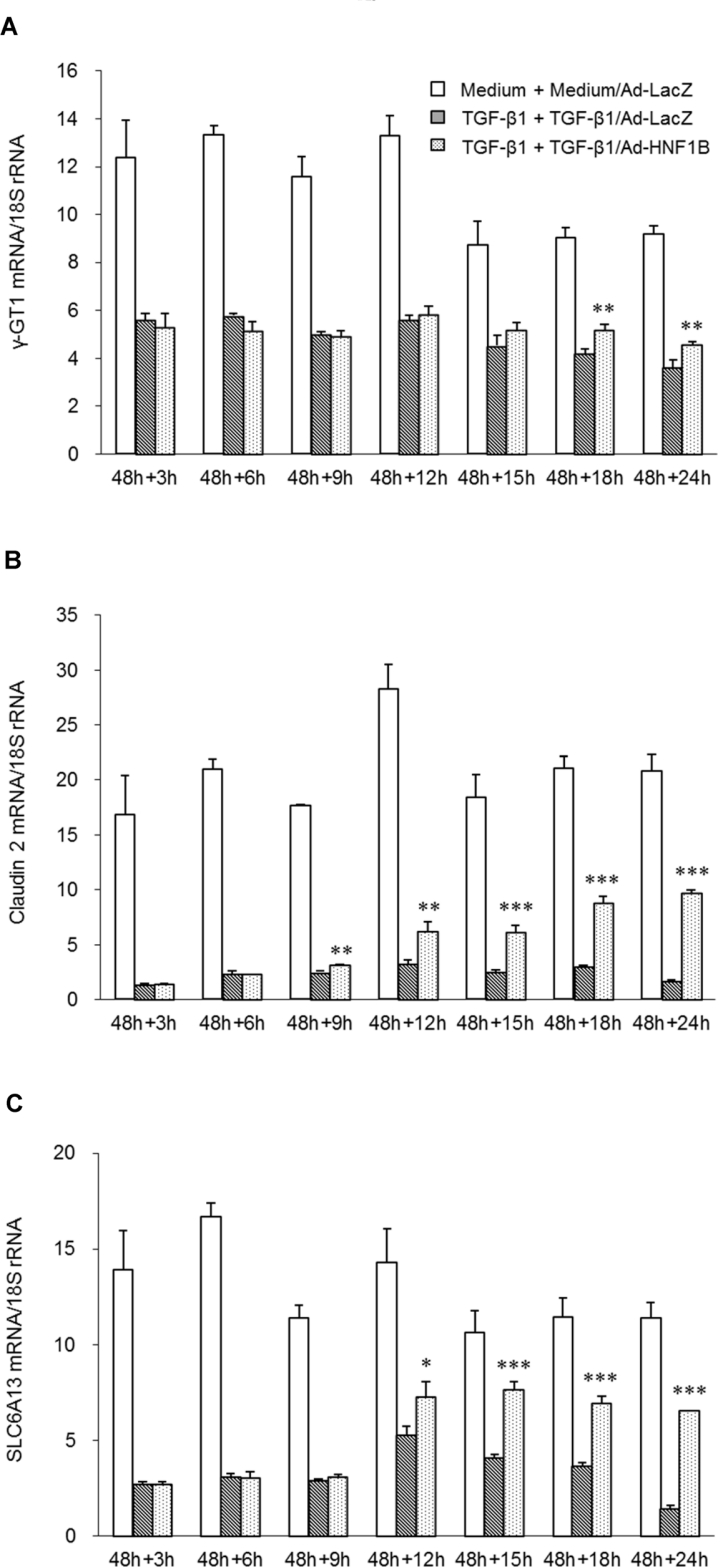
Time course of expression of proximal tubular epithelial marker genes in dedifferentiated hRPTECs after Ad-HNF1B infection. Human RPTECs were stimulated with 3 ng/ml TGF-β1 for 48 h, followed by re-stimulation with fresh TGF-β. After replacement with fresh TGF-β1, the hRPTECs were infected with 2.0 MOI Ad-HNF1B or Ad-LacZ. The levels of mRNA encoding γ-GT1 (A), claudin-2 (B), and SLC6A13 (C) in differentiated hRPTECs were determined by real-time RT-PCR analyses. Each column shows the data from medium incubation for 48 h followed by treatment with medium and Ad-LacZ (white), TGF-β1 stimulation for 48 h followed by treatment with TGF-β1 and Ad-LacZ (hatched line), and TGF-β1 and Ad-HNF1B for 24 h (dot). Each column and bar presents the means ± SD of three independent experiments. Statistical significance: * P < 0.05, ** P < 0.01, *** P < 0.001 vs. corresponding TGF-β1 and Ad-LacZ-treated groups by t-tests.

To further evaluate the action of HNF1B on the re-epithelialization process, the time course of gene expression during Ad-HNF1B-induced epithelialization was investigated. The epithelial marker genes, including γ-GT1, claudin-2, and SLC6A13, were downregulated by TGF-β1 stimulation for 48 h, and then began to increase significantly from 12 h to 18 h after Ad-HNF1B infection (Fig 6). The results suggest that re-epithelialization of the dedifferentiated hRPTECs was initiated at 12 h at the earliest after the infection. If HNF-1β indirectly induces the re-epithelialization mediated through HNF-1β downstream genes, the expression of the downstream gene is thought to change prior to the induction of epithelialization and after the upregulation of HNF-1β following Ad-HNF1B infection (Fig 3B and 3C). Therefore, to identify the HNF-1β downstream genes that induce re-epithelialization, the genes that were upregulated 9 h after Ad-HNF1B infection were analyzed by DNA microarray (n = 3) and real time RT-PCR. However, only a few genes, including NR1H4, SLCO4C1, EPHA7, and MITF, were found to have changed 9 h after the infection (S6 Fig), and the Ad-HNF1B-induced re-epithelialization represented by the normalization of both epithelial and mesenchymal marker genes was not affected by knockdown of any of the candidate genes (S7 Fig).

### Downregulation of HNF-1β expression in the renal fibrosis model

**Fig 7:**
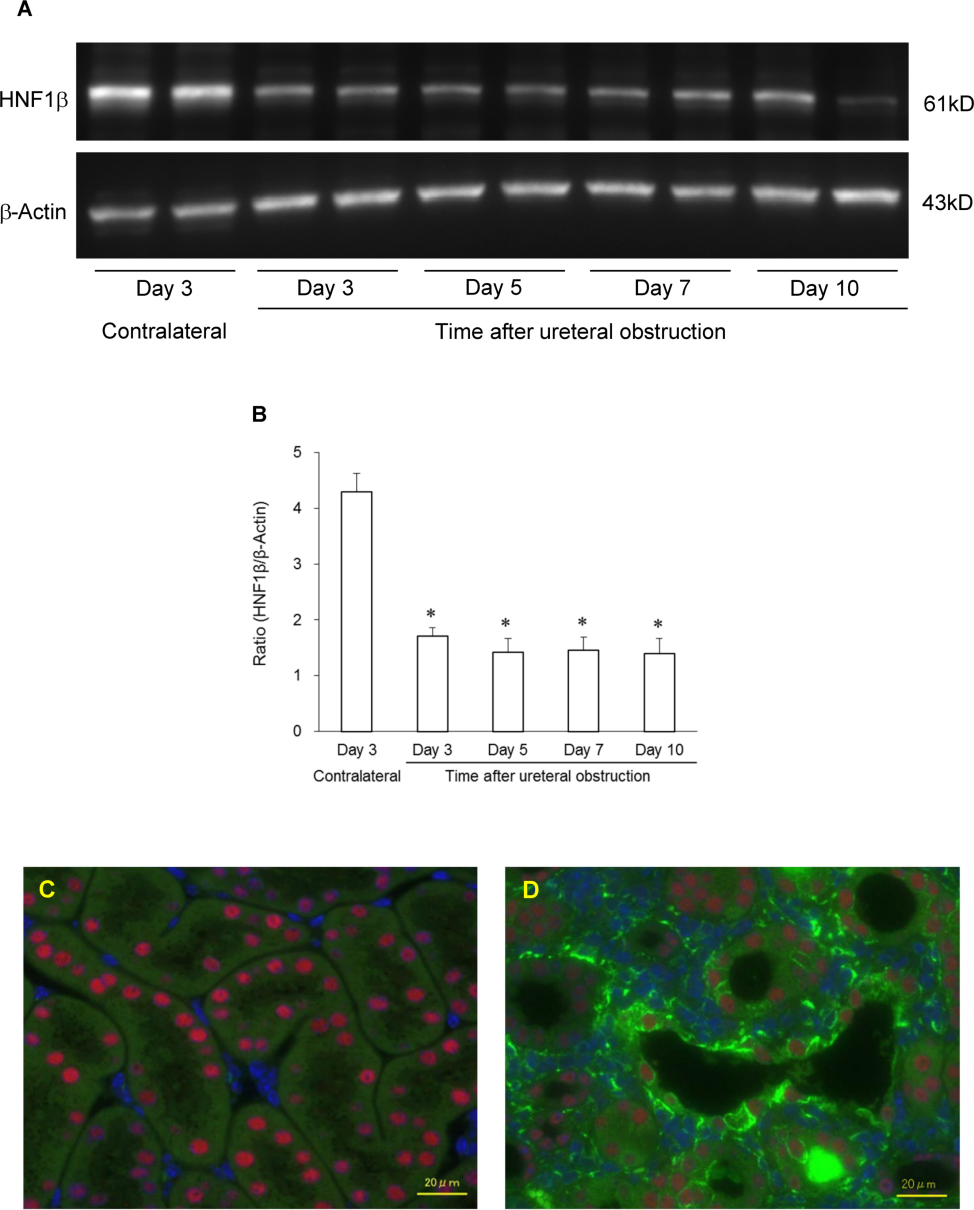
Expression of HNF-1β in mouse kidneys after ureteral obstruction. Representative western blotting shows the expression of HNF-1β at 61 kD, with β-actin as a control, in contralateral and obstructed kidneys (A). Densitometric quantification of the corresponding bands was performed using an image analyzer (B). The data are presented after normalization to β-actin expression. Each column and bar presents the mean ± SEM of four or five kidneys from UUO mice. Statistical significance was based on Wilcoxon’s rank-sum test; * *P* < 0.05 vs. contralateral kidney. Representative fluorescence microscopy photographs of renal sections represent HNF-1β and nestin protein expression in the contralateral non-obstructed kidney (C) and 7 days (D) after ureteral obstruction. Double-labeled immunofluorescence shows the expression of HNF1B (red) and nestin (green) using each specific antibody. The nuclei were counterstained with DAPI (blue). Scale bar = 20μm.

In mouse renal fibrosis models, renal fibrosis was significantly induced 5 days after ureteral obstruction compared with the contralateral kidney (S8 Fig). Expression of HNF-1β in the injured kidney began to decrease significantly 3 days after UUO (Fig 7A and 7B). The localization of HNF-1β was evaluated using immunohistological analysis. In the normal kidney, HNF-1β was expressed in the nuclei of most TECs, such as those of the proximal and distal tubules (Fig 7C). However, HNF-1β expression was suppressed in TECs 7 days after ureteral obstruction (Fig 7D). Furthermore, some of the HNF-1β downregulated TECs expressed mesenchymal marker, nestin and nestin-positive interstitial cells were also detected around the tubules in which the HNF-1β expression was suppressed (Fig 7D). The results suggest that the suppression of HNF-1β in TECs is associated with the dedifferentiation of TECs and the accumulation of the mesenchymal cells, including fibroblasts into the tubular interstitium in the kidney of in vivo renal fibrosis model.

## Discussions

Tubulointerstitial fibrosis is a major hallmark of all types of CKD and is involved in renal dysfunction [4,5]. We previously reported that an endogenous tetrapeptide N-acetyl-seryl-aspartyl-lysyl-proline that inhibited renal fibrosis via blockade of the profibrotic TGF-β signal transduction pathway significantly ameliorated the progression of renal dysfunction in a rat CKD model [19]. In tubulointerstitial injury, TECs are initially impaired by a variety of stimuli, including pathogenic microorganisms, inflammation, reactive oxygen species, chemical substances, and ischemic insults. It is accepted as an epithelial repair process in kidneys after acute injury that surviving cells adjacent to the impaired cells are dedifferentiated at first and migrate over the basement membrane, proliferate, and finally re-differentiate back into normal epithelial cells, resulting in the restoration of the nephron structure and function [20]. However, it has recently been recognized that acute kidney injury (AKI) in humans leads to acceleration of CKD progression, with tubulointerstitial fibrosis resulting from the maladaptive re-differentiation or persistent activation of the dedifferentiated TECs after AKI [21,22]. Rodent AKI models such as ischemia reperfusion and ureteral obstruction-induced injury have also shown dedifferentiation of TECs followed by severe tubular injury, leading to the development of interstitial fibrosis [23,24]. Recently, it was reported that the dedifferentiated TECs are arrested in the G2/M phase of the cell cycle, thus upregulating profibrotic mediator production, and that the circumvention of cell cycle arrest by pharmacological interventions attenuated the progression of several AKI models [12,25]. Another study showed that renal fibrosis in mice was remarkably reversed by treatment with bone morphogenetic protein 7, which induced the transition of the dedifferentiated phenotype to the normal epithelial status [26]. These reports suggest that dedifferentiated TECs play a crucial role in the development of renal fibrosis and that the re-epithelialization of dedifferentiated TECs lead to the improvement of the established renal fibrosis. In the present study, we showed that the dedifferentiated hRPTECs were restored to normal cells by removal of TGF-β1 from the culture medium. Using this re-epithelialization system, 48 genes were selected as candidate genes involved in re-epithelialization by means of DNA microarray analysis and biological network analysis (IPA). The functional validation study for the candidate genes showed that the re-epithelialization by TGF-β1 removal was abolished by HNF1B knockdown. The inhibitory activity of three types of siRNAs targeting HNF1B on epithelialization was closely correlated with their silencing strength for endogenous HNF1B gene expression. Although HNF-1α and HNF-1β share more than 90% homology in their DNA-binding domains and recognize the same target DNA-binding site [27], HNF1A knockdown did not affect the re-epithelialization. Patients with MODY (maturity onset diabetes of the young), which is caused by mutations in an autosomal dominant gene, are known to show distinct phenotypes between the HNF1B (MODY5) and HNF1A (MODY3) mutations due to differences in the expression level and the time and site of their expression [28]. The expression level of the HNF1B gene in hRPTECs that was measured in the present study was 10-fold higher than that of the HNF1A gene (data not shown). The difference between the effects of HNF1B and HNF1A knockdown for re-epithelialization may be due to their gene expression levels in hRPTECs. Furthermore, ectopic HNF-1β expression using Ad-HNF1B restored dedifferentiated hRPTECs to their normal state. HNF-1β has ever been reported to play an important role in the differentiation of renal TECs in the physiological environment and developmental stage. HNF-1β expression begins during the initial step of the developmental stage of the kidneys and continues to be expressed into adulthood, although this expression is then restricted to the epithelial cells [29,30]. Defective HNF-1β target genes caused by HNF1B mutation, including polycystin (Pkd2), polyductin (Pkhd1), and uromodulin (Umod), lead to the loss of orientation of the mitotic spindles in the renal epithelial cells during tubular elongation, resulting in the formation of renal cysts (polycystic kidney disease) [31]. In contrast, we found that HNF-1β induced the re-epithelialization of hRPTECs dedifferentiated by TGF-β1, which plays a pivotal role in the pathogenesis of renal fibrosis. Furthermore, the low dose (0.1 MOI and 0.3 MOI) of Ad-HNF1B induced the recovery of the downregulated epithelial marker genes but did not change the expression of the upregulated mesenchymal marker genes. This result suggests that HNF-1β may predominantly affect the epithelial marker genes rather than the mesenchymal marker genes, resulting in the re-epithelialization of the TECs that were dedifferentiated under pathological conditions.

We further evaluated the mechanism of action of HNF-1β in the re-epithelialization process. TGF-β1-induced dedifferentiation of epithelial cells is predominantly mediated by Smad3 activation [32]. However, the Smad3 phosphorylation in dedifferentiated hRPTECs is not inhibited by the ectopic expression of HNF-1β. The HNF-1β was downregulated by TGF-β stimulation for 48 h in the hRPTECs, and its expression began to increase 9 h after infection with Ad-HNF1B. Meanwhile, re-epithelialization, which is defined by the upregulation of epithelial marker genes, was induced at 12 h at the earliest after Ad-HNF1B infection. Accordingly, we believe that the expression of HNF-1β target genes should begin to change between 9 h (time of HNF-1β expression) and 12 h (time of re-epithelialization induction) after Ad-HNF1B infection. However, the expression of Pkd2, Pkhd1, Kif12, Cadherin16 and Socs3, which are known as HNF-1β target genes, did not change (data not shown), and only a few genes, including NR1H4, MITF, SLC3A1, and SLCO4C1, were significantly upregulated 9 h after Ad-HNF1B infection. From the functional validation analysis using four genes that target siRNAs, the HNF-1β-induced re-epithelialization was not affected by the knockdown of these genes. These results suggest that HNF-1β directly induced re-epithelialization independent of TGF-β1/Smad signaling since the HNF-1β-induced re-epithelialization was not mediated by the HNF-1β target genes, which were upregulated prior to the onset of epithelialization. The exact mechanism by which HNF-1β induces re-epithelialization still remains to be elucidated.

In the present study, we also showed that expression of the HNF-1β in the kidneys was suppressed in renal fibrosis model, which were elicited by UUO. The suppression of HNF-1β was observed prior to the significant development of the renal fibrosis. Histological study of the renal fibrosis model also showed the downregulation of the HNF-1β, which was expressed in all nephron segments of the normal kidneys. Furthermore, some of the HNF-1β-downregulated TECs expressed nestin and a large number of nestin-positive interstitial cells also accumulated around the HNF-1β-downregulated tubules. It has previously been reported that nestin is identified as a new marker of dedifferentiated TECs and activated fibroblast (myofibroblast) [13]. These results suggest that downregulation of HNF-1β in TECs are responsible for the dedifferentiation of TECs and the accumulation of scar-producing cells in the development of renal fibrosis. Wu *et al*. showed that cell cycle G2/M arrested TECs, which are dedifferentiated by TGF-β1, promoted the transition of pericytes/fibroblasts to scar-producing cells in a UUO renal fibrosis model [15]. In addition, Lan *et al*. reported that dedifferentiation of tubular epithelium was induced by persistent loss of PTEN (phosphatase and tensin homolog) in an ischemia-reperfusion renal fibrosis model and that the dedifferentiated TECs were surrounded by interstitial cells expressing vimentin and types I and III collagen [33]. It was further reported that the epithelial cell dedifferentiation and fibroblast activation in turn create a vicious cycle that leads to the acceleration of renal fibrosis and renal insufficiency [34]. From our results and previous reports, we concluded that HNF-1β suppression in dedifferentiated tubules is a crucial event for the dedifferentiation of TECs and that the upregulation of HNF-1β in the dedifferentiated TECs can restore the dedifferentiated TECs into their normal state, which leads to the attenuation of the TECs-fibroblasts interaction, and then quiescence of the activated fibroblasts, thus eventually alleviating renal fibrosis. Although the inhibitory effect of HNF-1β upregulation on renal fibrosis progression remains to be elucidated, the re-epithelialization by regulation of HNF-1β may represent an epochal therapeutic strategy for renal fibrosis.

## Supporting Information

S1 Fig Biological network extracted by Ingenuity Pathway Analysis among the genes whose expression differentially changed during re-epithelialization after TGF-β1 removal. The list of genes whose expression differed by more than twofold between re-epithelialized hRPTECs 24 h after TGF-β1 removal and dedifferentiated hRPTECs 24 h after TGF-β1 re-stimulation for 24 h were analyzed by Ingenuity Pathway Analysis. Representative networks involved in re-epithelialization show four biological signaling networks in which HNF1A/B (A), CEBPA (B), BCL2 (C), and HNF4A (D) were centrally located.
(TIFF)

S2 Fig Time course of candidate gene expression involved in re-epithelialization by TGF-β1 removal. Human RPTECs were cultivated with medium or 3 ng/ml TGF-β1 for 48 h followed by incubation in fresh medium with or without TGF-β1 for 24 h. The expression changes of representative candidate genes (A: HNF1A, B: HNF1B, C: HNF4A, D: LGALS2, E: GDF15, F: NAT8, G: ELF3, H: CEBPA, and I: NR1H4) within 24 h after the removal of TGF-β1 were determined by real-time RT-PCR. Each column shows data from non-stimulation for 48 h (white), TGF-β1 stimulation for 48 h followed by TGF-β1 re-stimulation (gray), and TGF-β1 stimulation for 48 h followed by incubation with TGF-β1-free fresh medium (dot). Each column and bar presents the means ± SD of three independent experiments. Statistical significance: # P < 0.05, ## P < 0.01, ### P < 0.001 vs. medium group (white); * P < 0.05, ** P < 0.01, *** P < 0.001 vs. TGF-β1 re-stimulation group (gray) at each time point by t-tests.
(TIFF)

S3 Fig Effects of candidate gene-targeting siRNA on the expression changes of epithelial and mesenchymal marker genes in re-epithelialized hRPTECs by TGF-β1 removal. Human RPTECs were cultivated with medium or 3 ng/ml TGF-β1 for 48 h, followed by incubation in fresh medium with or without TGF-β1 for 48 h. Cells were treated with three types of siRNA (15 nM) for each candidate gene (HNF1A, HNF1B, HNF4A, LGALS2, GDF15, and NAT8) and two types of siRNA for negative control (Control) (15 nM) for 24 h after the first TGF-β1 stimulation. The levels of mRNA encoding proximal tubular epithelial marker genes (A: γ-GT1 and B: claudin-2) and mesenchymal marker genes (C: type I collagen and D: fibronectin) were determined by real-time RT-PCR analyses. Each column presents the means of twice experiments for siRNA-1 (white), siRNA-2 (gray), and siRNA-3 (dot). Each dot symbol shows an individual value. The dotted line indicates the gene expression in control siRNA-treated groups that were first stimulated with TGF-β1, followed by incubation with TGF-β1-free medium.
(TIFF)

S4 Fig Effects of candidate gene–targeting siRNA on the expression of corresponding endogenous mRNA in TGF-β1-stimulated hRPTECs. Human RPTECs were cultivated with medium or 3 ng/ml TGF-β1 for 48 h, followed by incubation in fresh medium with or without TGF-β1 for 48 h. Cells were treated with three types of siRNA (15 nM) for each candidate gene and two types of siRNA for negative control (Control) (15 nM) for 24 h after onset of TGF-β1 stimulation. The levels of mRNA encoding the candidate genes (A: HNF1A, B: HNF1B, C: HNF4A, D: LGALS2, E: GDF15, and F: NAT8) were determined by real-time RT-PCR analyses. Each column presents the means of twice experiments for siRNA-1 (white), siRNA-2 (gray), and siRNA-3 (dot). Each dot symbol shows an individual value.
(TIFF)

S5 Fig Effect of HNF1B siRNA on the re-epithelialization of dedifferentiated hRPTECs by Ad-HNF1B infection. Human RPTECs were stimulated with 3 ng/ml TGF-β1 for 48 h, followed by re-stimulation with fresh TGF-β1 for 72 h. After replacement with fresh TGF-β1, hRPTECs were infected with 2.0 MOI Ad-HNF1B or Ad-LacZ. Cells were treated with HNF1B-targeting siRNA (15 nM) and negative control siRNA (Control) (15 nM) for 24 h after the first TGF-β1 stimulation. The levels of mRNA encoding γ-GT1 (A), claudin-2 (B), type I collagen (C), and fibronectin (D) in the differentiated hRPTECs were determined by real-time RT-PCR. Each column presents the means of twice experiments for medium/Ad-LacZ + control siRNA (white), TGF-β/Ad-LacZ + control siRNA (gray), TGF-β/Ad-HNF1B + control siRNA (light gray), and TGF-β/Ad-HNF1B + HNF1B siRNA (black). Each dot symbol shows an individual value.
(TIFF)

S6 Fig Expression of HNF-1β downstream genes in dedifferentiated hRPTECs 9 h after Ad-HNF1B infection. Human RPTECs were stimulated with 3 ng/ml TGF-β1 for 48 h, followed by re-stimulation with fresh TGF-β for 9 h. After replacement with fresh TGF-β1, the hRPTECs were infected with 2.0 MOI Ad-HNF1B or Ad-LacZ. The levels of mRNA encoding EPHA7 (A), SLCO4C1 (B), NR1H4 (C), and MITF (D) in differentiated hRPTECs were determined by real-time RT-PCR analyses. Each column shows data from non-stimulation (white), TGF-β1 stimulation for 48 h (gray), TGF-β1 stimulation for 48 h followed by treatment with TGF-β1 and Ad-LacZ for 9 h (hatched line), and TGF-β1 and Ad-HNF1B (dot) for 9 h. Each column and bar presents the mean ± SD from three independent experiments. Each dot symbol shows the mean from each experiment. Statistical significance: ** *P* < 0.01 vs. corresponding TGF-β1- and Ad-LacZ-treated group (dotted column); # *P* < 0.05, ## *P* < 0.01 vs. 48-h TGF-β1-treated group (gray column) by *t*-tests.
(TIFF)

S7 Fig Effects of HNF-1β downstream gene siRNA on re-epithelialization of dedifferentiated hRPTECs by Ad-HNF1B infection. Human RPTECs were stimulated with 3 ng/ml TGF-β1 for 48 h, followed by re-stimulation with fresh TGF-β1 for 72 h. After replacement with fresh TGF-β1, the hRPTECs were infected with 2.0 MOI Ad-HNF1B or Ad-LacZ. Cells were treated with three types of siRNA (15 nm) for each HNF-1β downstream gene (EPHA7, SLCO4A1, NR1H4, and MITF) and two types of siRNA for negative control (Control) (15 nm) for 24 h after the first TGF-β1 stimulation. The levels of mRNA encoding γ-GT1 (A), claudin-2 (B), type I collagen (C), and fibronectin (D) in the differentiated hRPTEC were determined by real-time RT-PCR. Each column presents the means of twice experiments for siRNA-1 (white), siRNA-2 (gray), and siRNA-3 (dot). The dotted line indicates the gene expression in TGF-β/Ad-HNF1B + control siRNA. Each dot symbol shows an individual value.
(TIFF)

S8 Fig Time course for development of renal interstitial fibrosis in mice with unilateral ureteral obstruction. Representative Masson’s trichrome-stained photomicrographs of tubular lesions in a contralateral unobstructed kidney (A) and obstructed kidneys 3 days (B), 5 days (C), 7 days (D), and 10 days (E) after unilateral ureteral obstruction. Scale bar = 40 μm. The percentage of the fibrotic area that was stained blue was calculated relative to the entire field area (F). Each column and bar presents the mean ± SEM of five kidneys at each test day after unilateral ureteral obstruction. Each dot symbol shows an individual value. Statistical significance: *** P < 0.001 vs. contralateral kidney by Dunnett’s test.
(TIF)

S1 Table List of genes upregulated during re-epithelialization. Gene expression profiling of the dedifferentiated hRPTECs that were stimulated by TGF-β1 for 48 h (Group 1) and followed by additional TGF-β1 stimulation for 24 h (Group 2) and the re-epithelialized hRPTECs by TGF-β1 removal (Group 3) was investigated by DNA microarray. Comparative differential gene expression analysis among these three groups using the Subio Platform revealed that 872 genes were downregulated in Group 2 compared with Group 1 and were upregulated more than two-fold in Group 3 compared with Group 1.
(ZIP)

S2 Table List of candidate genes involved in induction of re-epithelialization. Biological networks of genes altered during re-epithelialization by TGF-β1 removal were analyzed using IPA. Forty-eight genes that were located in central position of 25 networks generated through IPA were selected as candidate genes involved in re-epithelialization.
(XLSX)

## Abbreviations

HNF1A: hepatocyte nuclear factor 1 homeobox A
HNF1B: hepatocyte nuclear factor 1 homeobox B
HNF4A: hepatocyte nuclear factor 4A
LGALS2: lectin, galactoside-binding, soluble, 2
GDF15: growth differentiation factor 15
NAT8: N-acetyltransferase 8 (GCN5-related, putative)
EPHA7: Ephrin type-A receptor 7
SLCO4A1: solute carrier organic anion transporter family member 4A1
NR1H4: nuclear receptor subfamily 1, group H, member 4
CEBPA: CCAAT/enhancer binding protein (C/EBP), alpha
ELF3: E74-like factor 3
SLC6A13: solute carrier family 6 (neurotransmitter transporter), member 13
Kif2: kinesin family member 12
IPA: Ingenuity Pathway Analysis
