# Supplementary figures and images for: Hepatocyte Nuclear Factor-1β Induces Redifferentiation of Dedifferentiated Tubular Epithelial Cells
Source: PLoS One. 2016 May 19;11(5):e0154912. doi: 10.1371/journal.pone.0154912 (PMC4873210; doi:10.1371/journal.pone.0154912)

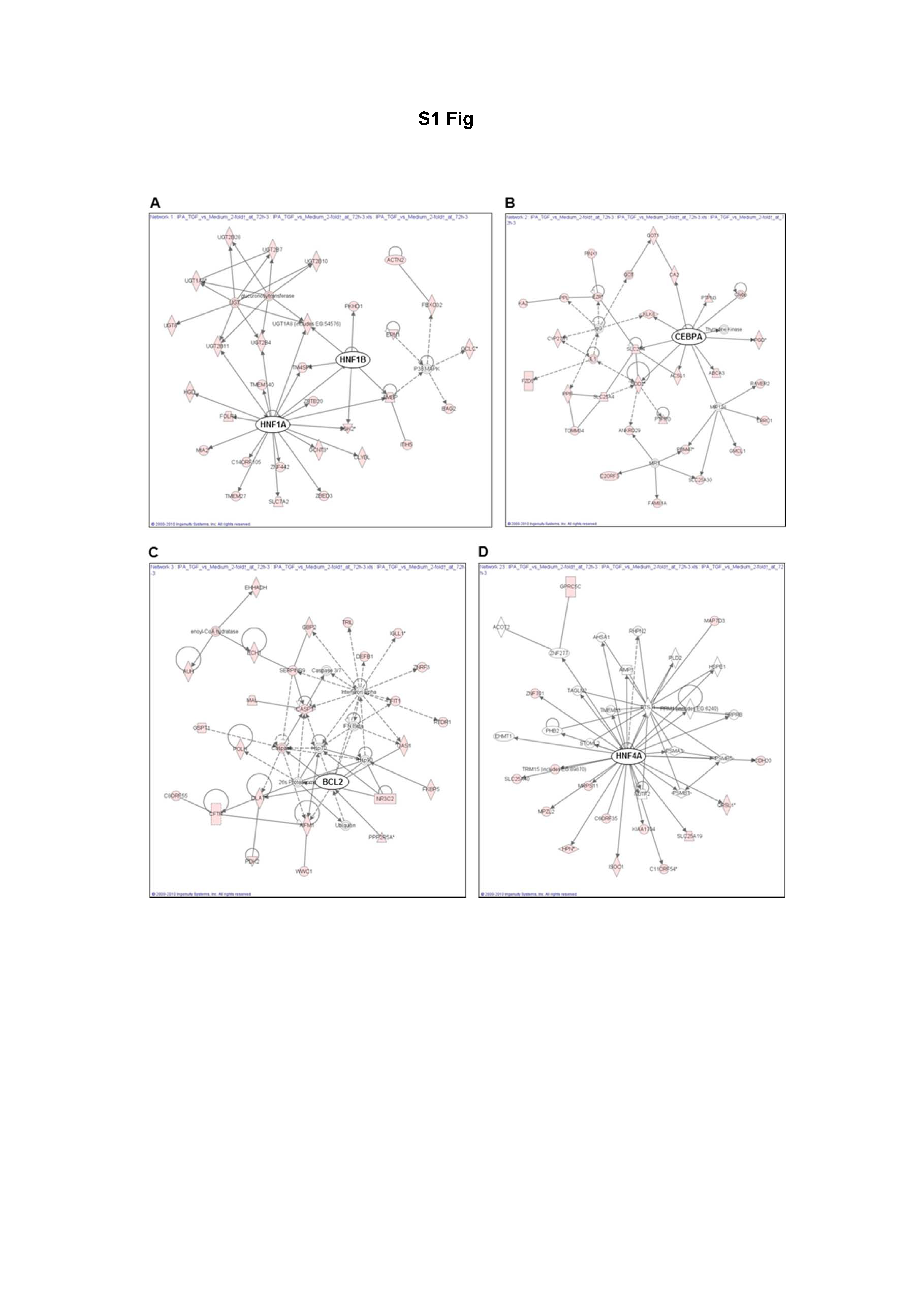

Supplement: S1 Fig — The list of genes whose expression differed by more than twofold between re-epithelialized hRPTECs 24 h after TGF-β1 removal and dedifferentiated hRPTECs 24 h after TGF-β1 re-stimulation for 24 h were analyzed by Ingenuity Pathway Analysis. Representative networks involved in re-epithelialization show four biological signaling networks in which HNF1A/B (A), CEBPA (B), BCL2 (C), and HNF4A (D) were centrally located. (TIFF) [file pone.0154912.s001.tiff]

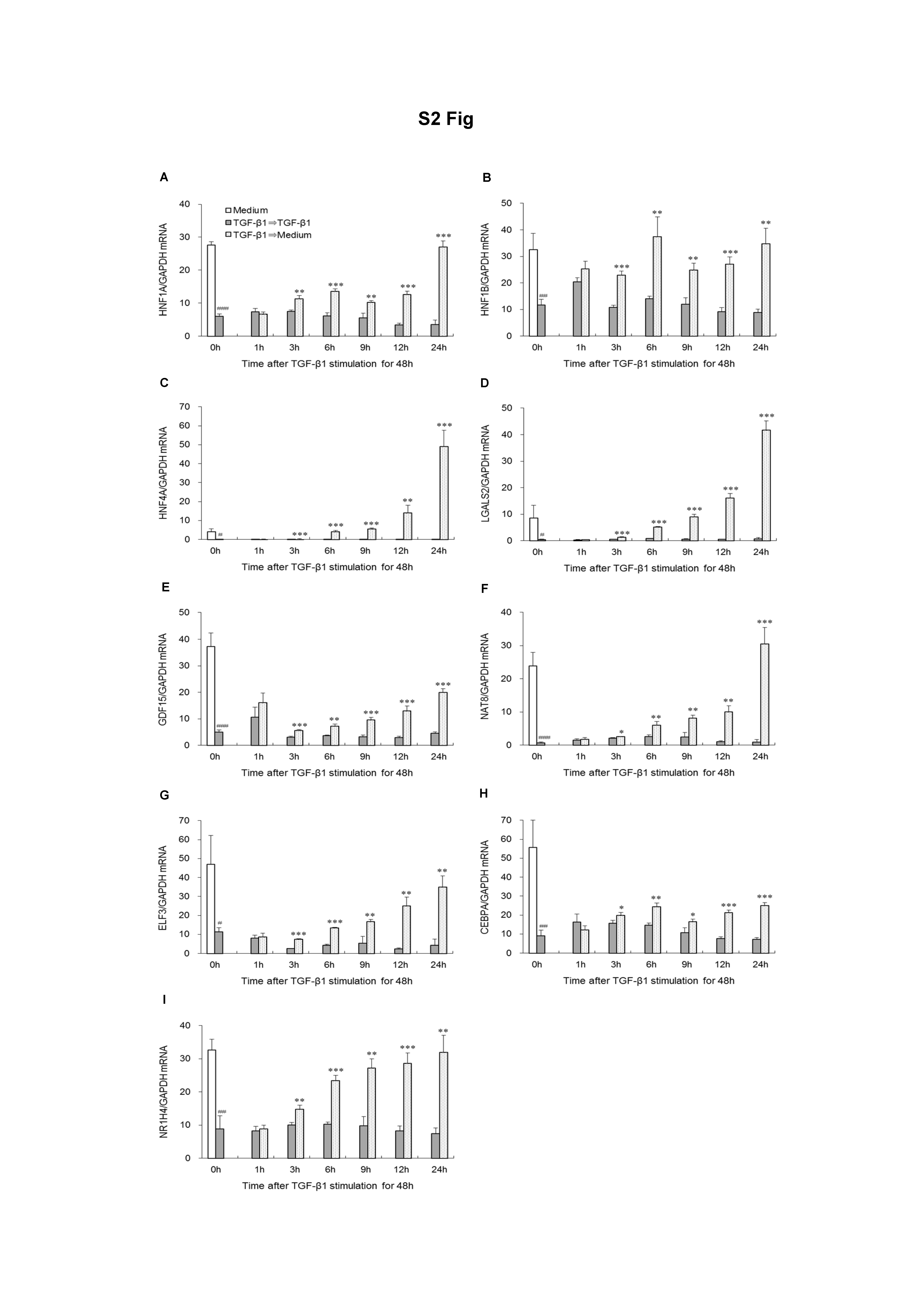

Supplement: S2 Fig — Human RPTECs were cultivated with medium or 3 ng/ml TGF-β1 for 48 h followed by incubation in fresh medium with or without TGF-β1 for 24 h. The expression changes of representative candidate genes (A: HNF1A, B: HNF1B, C: HNF4A, D: LGALS2, E: GDF15, F: NAT8, G: ELF3, H: CEBPA, and I: NR1H4) within 24 h after the removal of TGF-β1 were determined by real-time RT-PCR. Each column shows data from non-stimulation for 48 h (white), TGF-β1 stimulation for 48 h followed by TGF-β1 re-stimulation (gray), and TGF-β1 stimulation for 48 h followed by incubation with TGF-β1-free fresh medium (dot). Each column and bar presents the means ± SD of three independent experiments. Statistical significance: # P < 0.05, ## P < 0.01, ### P < 0.001 vs. medium group (white); * P < 0.05, ** P < 0.01, *** P < 0.001 vs. TGF-β1 re-stimulation group (gray) at each time point by t-tests. (TIFF) [file pone.0154912.s002.tiff]

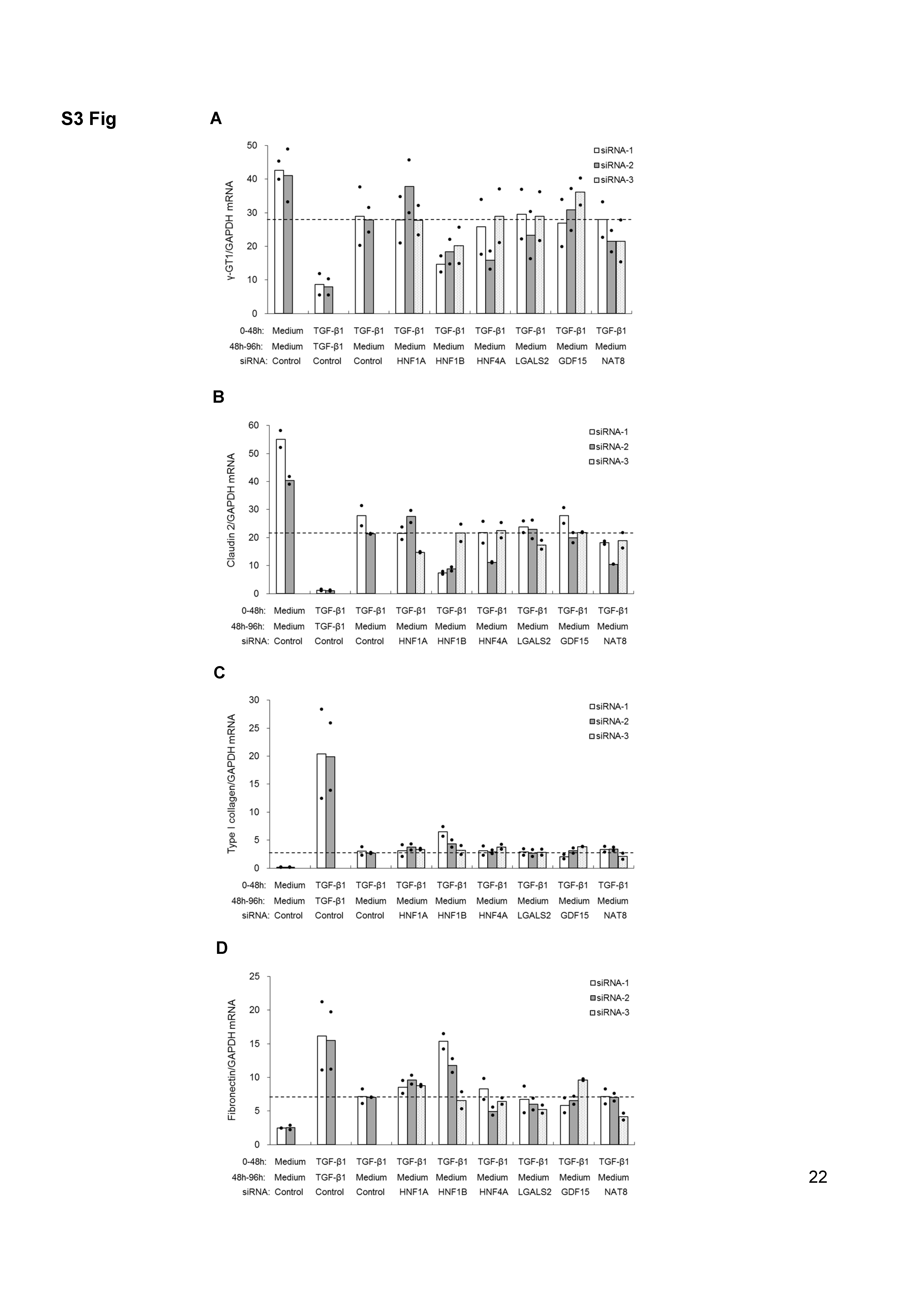

Supplement: S3 Fig — Human RPTECs were cultivated with medium or 3 ng/ml TGF-β1 for 48 h, followed by incubation in fresh medium with or without TGF-β1 for 48 h. Cells were treated with three types of siRNA (15 nM) for each candidate gene (HNF1A, HNF1B, HNF4A, LGALS2, GDF15, and NAT8) and two types of siRNA for negative control (Control) (15 nM) for 24 h after the first TGF-β1 stimulation. The levels of mRNA encoding proximal tubular epithelial marker genes (A: γ-GT1 and B: claudin-2) and mesenchymal marker genes (C: type I collagen and D: fibronectin) were determined by real-time RT-PCR analyses. Each column presents the means of twice experiments for siRNA-1 (white), siRNA-2 (gray), and siRNA-3 (dot). Each dot symbol shows an individual value. The dotted line indicates the gene expression in control siRNA-treated groups that were first stimulated with TGF-β1, followed by incubation with TGF-β1-free medium. (TIFF) [file pone.0154912.s003.tiff]

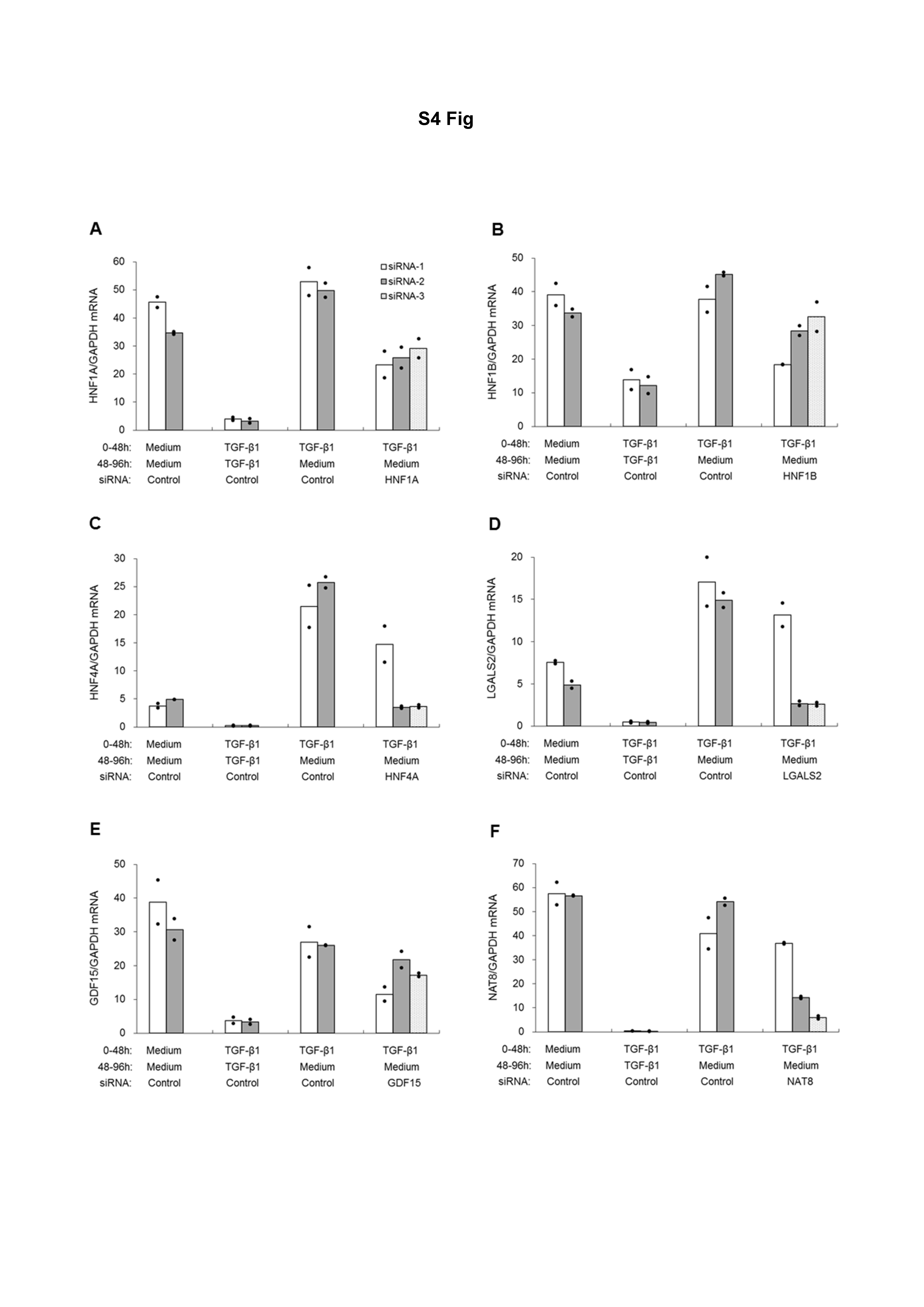

Supplement: S4 Fig — Human RPTECs were cultivated with medium or 3 ng/ml TGF-β1 for 48 h, followed by incubation in fresh medium with or without TGF-β1 for 48 h. Cells were treated with three types of siRNA (15 nM) for each candidate gene and two types of siRNA for negative control (Control) (15 nM) for 24 h after onset of TGF-β1 stimulation. The levels of mRNA encoding the candidate genes (A: HNF1A, B: HNF1B, C: HNF4A, D: LGALS2, E: GDF15, and F: NAT8) were determined by real-time RT-PCR analyses. Each column presents the means of twice experiments for siRNA-1 (white), siRNA-2 (gray), and siRNA-3 (dot). Each dot symbol shows an individual value. (TIFF) [file pone.0154912.s004.tiff]

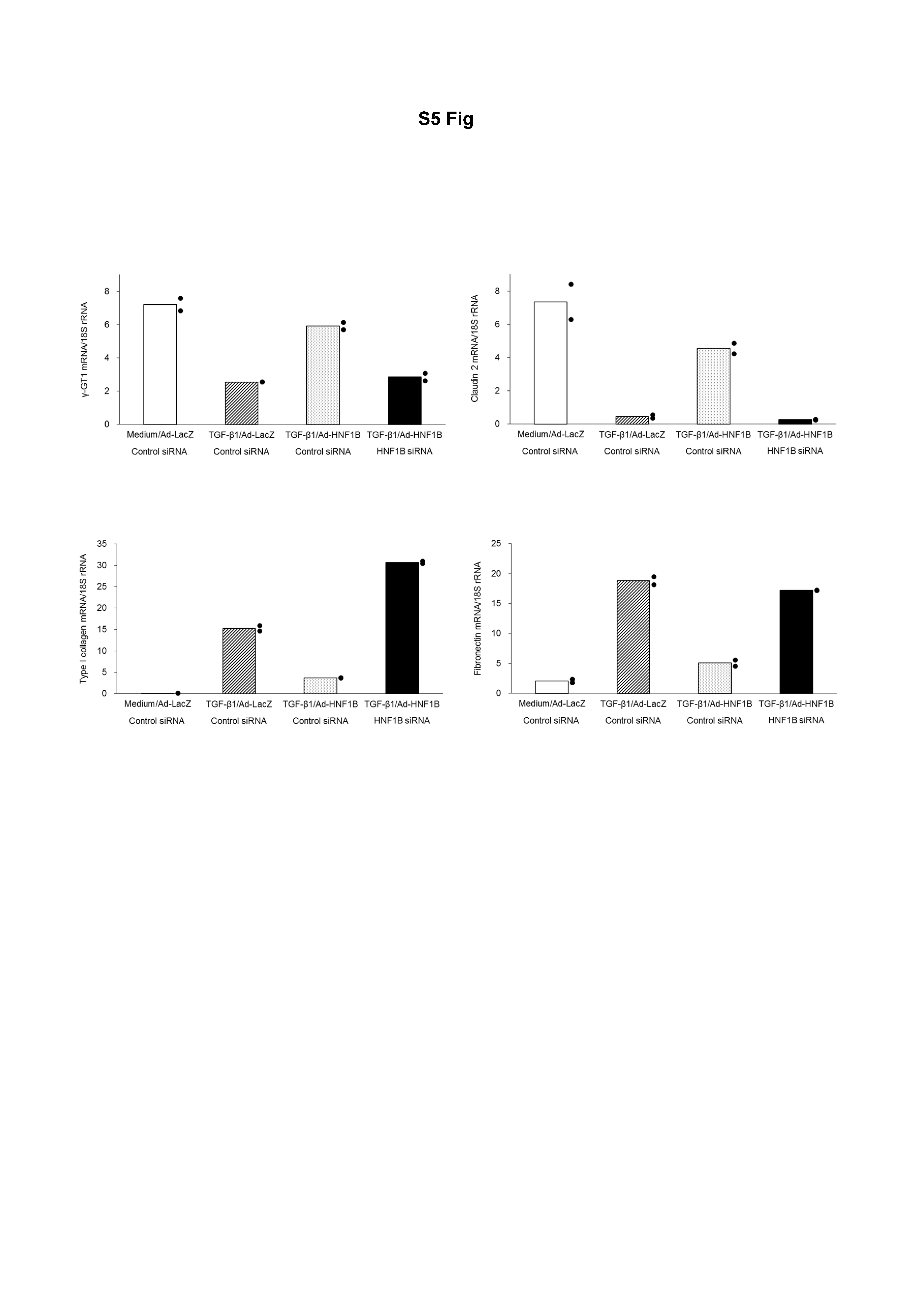

Supplement: S5 Fig — Human RPTECs were stimulated with 3 ng/ml TGF-β1 for 48 h, followed by re-stimulation with fresh TGF-β1 for 72 h. After replacement with fresh TGF-β1, hRPTECs were infected with 2.0 MOI Ad-HNF1B or Ad-LacZ. Cells were treated with HNF1B-targeting siRNA (15 nM) and negative control siRNA (Control) (15 nM) for 24 h after the first TGF-β1 stimulation. The levels of mRNA encoding γ-GT1 (A), claudin-2 (B), type I collagen (C), and fibronectin (D) in the differentiated hRPTECs were determined by real-time RT-PCR. Each column presents the means of twice experiments for medium/Ad-LacZ + control siRNA (white), TGF-β/Ad-LacZ + control siRNA (gray), TGF-β/Ad-HNF1B + control siRNA (light gray), and TGF-β/Ad-HNF1B + HNF1B siRNA (black). Each dot symbol shows an individual value. (TIFF) [file pone.0154912.s005.tiff]

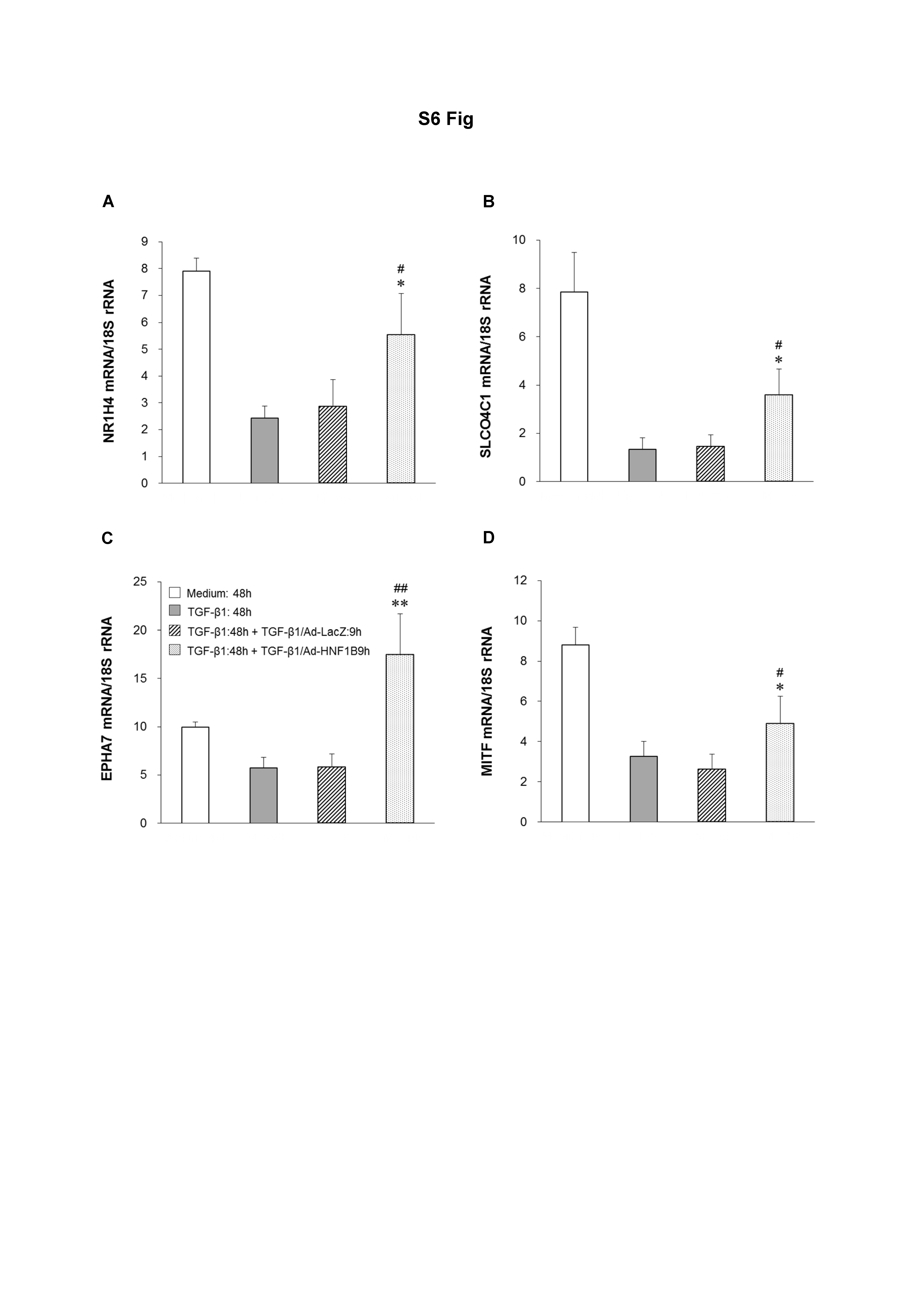

Supplement: S6 Fig — Human RPTECs were stimulated with 3 ng/ml TGF-β1 for 48 h, followed by re-stimulation with fresh TGF-β for 9 h. After replacement with fresh TGF-β1, the hRPTECs were infected with 2.0 MOI Ad-HNF1B or Ad-LacZ. The levels of mRNA encoding EPHA7 (A), SLCO4C1 (B), NR1H4 (C), and MITF (D) in differentiated hRPTECs were determined by real-time RT-PCR analyses. Each column shows data from non-stimulation (white), TGF-β1 stimulation for 48 h (gray), TGF-β1 stimulation for 48 h followed by treatment with TGF-β1 and Ad-LacZ for 9 h (hatched line), and TGF-β1 and Ad-HNF1B (dot) for 9 h. Each column and bar presents the mean ± SD from three independent experiments. Each dot symbol shows the mean from each experiment. Statistical significance: ** P < 0.01 vs. corresponding TGF-β1- and Ad-LacZ-treated group (dotted column); # P < 0.05, ## P < 0.01 vs. 48-h TGF-β1-treated group (gray column) by t-tests. (TIFF) [file pone.0154912.s006.tiff]

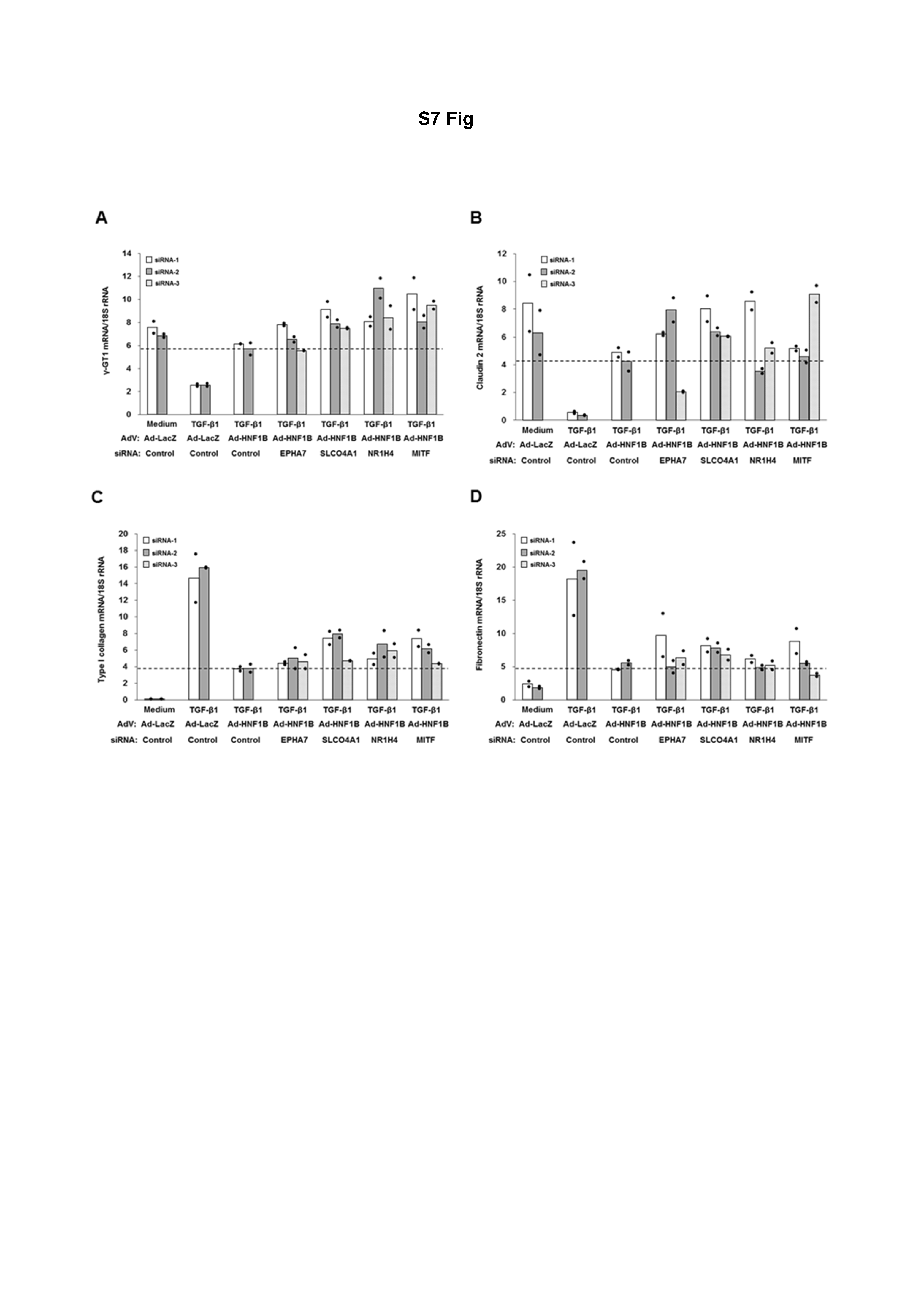

Supplement: S7 Fig — Human RPTECs were stimulated with 3 ng/ml TGF-β1 for 48 h, followed by re-stimulation with fresh TGF-β1 for 72 h. After replacement with fresh TGF-β1, the hRPTECs were infected with 2.0 MOI Ad-HNF1B or Ad-LacZ. Cells were treated with three types of siRNA (15 nm) for each HNF-1β downstream gene (EPHA7, SLCO4A1, NR1H4, and MITF) and two types of siRNA for negative control (Control) (15 nm) for 24 h after the first TGF-β1 stimulation. The levels of mRNA encoding γ-GT1 (A), claudin-2 (B), type I collagen (C), and fibronectin (D) in the differentiated hRPTEC were determined by real-time RT-PCR. Each column presents the means of twice experiments for siRNA-1 (white), siRNA-2 (gray), and siRNA-3 (dot). The dotted line indicates the gene expression in TGF-β/Ad-HNF1B + control siRNA. Each dot symbol shows an individual value. (TIFF) [file pone.0154912.s007.tiff]

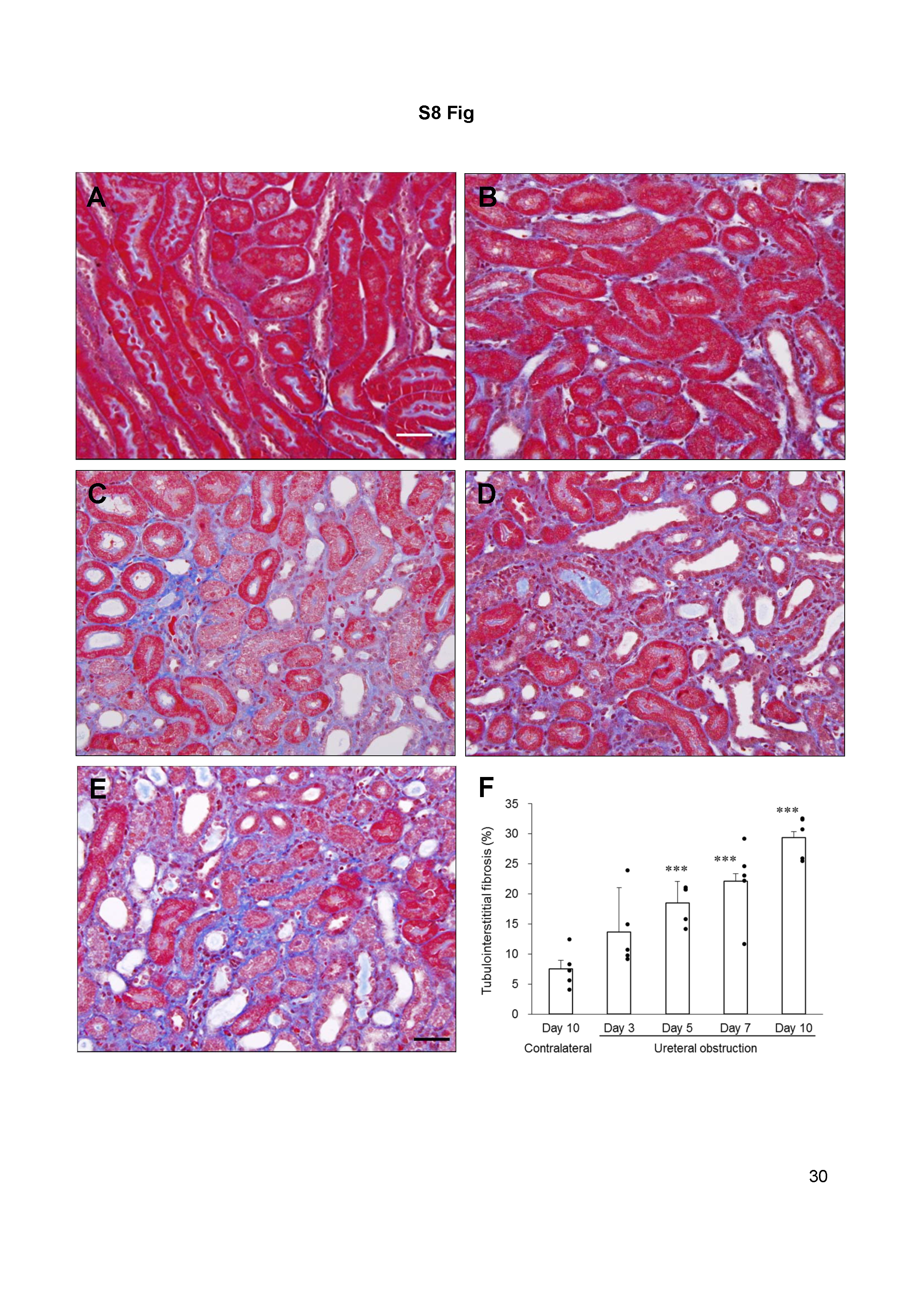

Supplement: S8 Fig — Representative Masson’s trichrome-stained photomicrographs of tubular lesions in a contralateral unobstructed kidney (A) and obstructed kidneys 3 days (B), 5 days (C), 7 days (D), and 10 days (E) after unilateral ureteral obstruction. Scale bar = 40 μm. The percentage of the fibrotic area that was stained blue was calculated relative to the entire field area (F). Each column and bar presents the mean ± SEM of five kidneys at each test day after unilateral ureteral obstruction. Each dot symbol shows an individual value. Statistical significance: *** P < 0.001 vs. contralateral kidney by Dunnett’s test. (TIF) [file pone.0154912.s008.tif]
